# Supplementary material for: Somatic mTOR mutation in clonally expanded T lymphocytes associated with chronic graft versus host disease
Source: Nat Commun. 2020 May 7;11:2246. doi: 10.1038/s41467-020-16115-w (PMC7206083; doi:10.1038/s41467-020-16115-w)
Supplement: Supplementary file 1 — Supplementary Information [file 41467_2020_16115_MOESM1_ESM.pdf]

Supplementary Information

Somatic mTOR mutation in clonally expanded T lymphocytes associated with chronic graft versus host disease

Kim et al.

Supplementary Appendix

Table of contents

Supplementary results page 3-5

Supplementary Figures

Supplementary Figure 1 page 6
Supplementary Figure 2 page 7-10
Supplementary Figure 3 page 11
Supplementary Figure 4 page 12-13
Supplementary Figure 5 page 14-15
Supplementary Figure 6 page 16-17
Supplementary Figure 7 page 18-19
Supplementary Figure 8 page 20-24
Supplementary Figure 9 page 25-26
Supplementary Figure 10 page 27
Supplementary Figure 11 page 28

Supplementary Tables

Supplementary Table 1 page 29-30
Supplementary Table 2 page 31
Supplementary Table 3 page 32
Supplementary Table 4 page 33
Supplementary Table 5 page 34
Supplementary Table 6 page 35-36
Supplementary Table 7 page 37-38
Supplementary Table 8 page 39
Supplementary Table 9 page 40
Supplementary Table 10 page 41
Supplementary Table 11 page 42
Supplementary Table 12 page 43

Supplementary results

Clinical Characteristics of the Index cGvHD Patient 1

The index patient was a 56-year-old male, who was diagnosed with chronic phase chronic myeloid leukemia (CML) in the fall 1999. The clinical status and treatment history are described in detail in Supplemental Figure 1. After short leukocyte reduction with hydroxyurea the patient received allo-HSCT from his HLA-matched sibling (male) donor after myeloablative conditioning. Double immunosuppression with cyclosporine and methylprednisone was started at the time of the allo-HSCT, and methotrexate was also given on days +1, +3 and +6 after the transplantation. Within the first 100 days the patient had a human herpesvirus 6 (HHV6) infection and the first cytomegalovirus (CMV) reactivation. At the end of that period, the first symptoms of chronic GvHD emerged in the form of elevated transaminases. Since the beginning of 2001, the patient suffered from cGvHD affecting his liver, eyes, nails and skin including sclerodermatous skin lesions that presented with a varying degree of severity over time. Mycophenolate was added to the immunosuppressive regimen in the spring 2001. In the course of the cGvHD, the patient was treated with photopheresis in 2002 as well as with a low-dose irradiation of his lymph nodes in 2003. The immunosuppression was continuously adjusted according to the clinical presentation of the cGvHD. In recent years, chronic slerodermatous skin lesions, fluctuating liver enzymes and cGvHD of the eyes have persisted and required constant treatment. The patient has continuously received mycophenolate and varying supplementation with methylprednisone and cyclosporine. The patient has never had sirolimus or everolimus therapy. The patient has not had a relapse of CML nor received any donor lymphocyte infusions. He was sampled (blood) for the first time in 2013 and in the disease course thereafter (Supplementary Figure 1).

Clinical Characteristics of the 2nd cGvHD Patient with *mTOR* mutation

The patient 2 was a 66-year-old woman with AML/MDS. The patient received allo-HSCT from HLA-matched non-related (female) donor after reduced intensity conditioning early in 2013. The graft-source was mobilized peripheral blood stem cells. The primary prophylaxis for GVHD were cyclosporine and metotrexate. The patient did not suffer from acute GVHD. 9 months after the transplantation she developed a cutaneous and ocular chronic GVHD. Prednison was initiated alongside with cyclosporine. Chronic nephrotic syndrome (membranous glomerulonephritis (MGN) as a manifestation of cGvHD) with 9 gr/day of proteinuria developed three years after the transplantation in the winter 2016. The *mTOR P2229R* mutation was detected in a peripheral blood sample that was taken a month later. MGN resolved with corticosteroid therapy. Only one blood sample from the patient was available, and thus disappearance or persistence of the mutation could not be confirmed. Chronic ocular GvHD has persisted over the years. The patient has not had a relapse nor received any donor lymphocyte infusions.

Clinical Characteristics of the 3rd cGvHD Patient with *mTOR* mutation

The patient 3 was a 48-year-old woman with AML. The patient received allo-HSCT from her HLA-matched sibling (male) donor after myeloablative conditioning in August 2014. The graft source was mobilized peripheral blood stem cells. The primary prophylaxis for GvHD were cyclosporine and metotrexate. The patient did not have any CMV reactivation nor onset of acute GvHD after the transplantation. Cyclosporine was tapered by 4.5 months. A month later the disease relapsed in the bone marrow, and the patient got re-induction with fludarabine, high dose cytarabine, G-CSF and idarubicine (FLAG-IDA). Full donor chimerism was achieved. Three months later the patient developed extramedullary skin relapse (bone marrow was in complete remission) that was successfully treated with

two donor lymphocyte infusions and azacytidine. Three months later the patient developed moderate cGvHD affecting skin, mouth, eyes and liver. Also, eosinophilia, thrombocytopenia and hypergammaglobulinemia were detected. The *mTOR P2229R* mutation was detected in a peripheral blood sample concurrently. The initial treatment was prednisone 1mg/kg/d. During the following four months partial response was achieved after which prednisone was tapered by 20 % a month. In the following four months, prednisone was completely tapered, and no cGvHD was present. Thrombocytopenia recovered, but liver enzymes were elevated probably due to toxic effect. However, cGvHD of the liver could not be excluded. A month later the patient developed a second extramedullary relapse involving CNS. Intrathecal chemotherapy and supportive therapy were given, but the patient succumbed in two months. Patient provided multiple blood samples before and after the onset of cGvHD. The *mTOR P2229R* mutation was detected in a sample taken after the onset of cGvHD.

Supplementary Figure 1. Medical history of the index patient

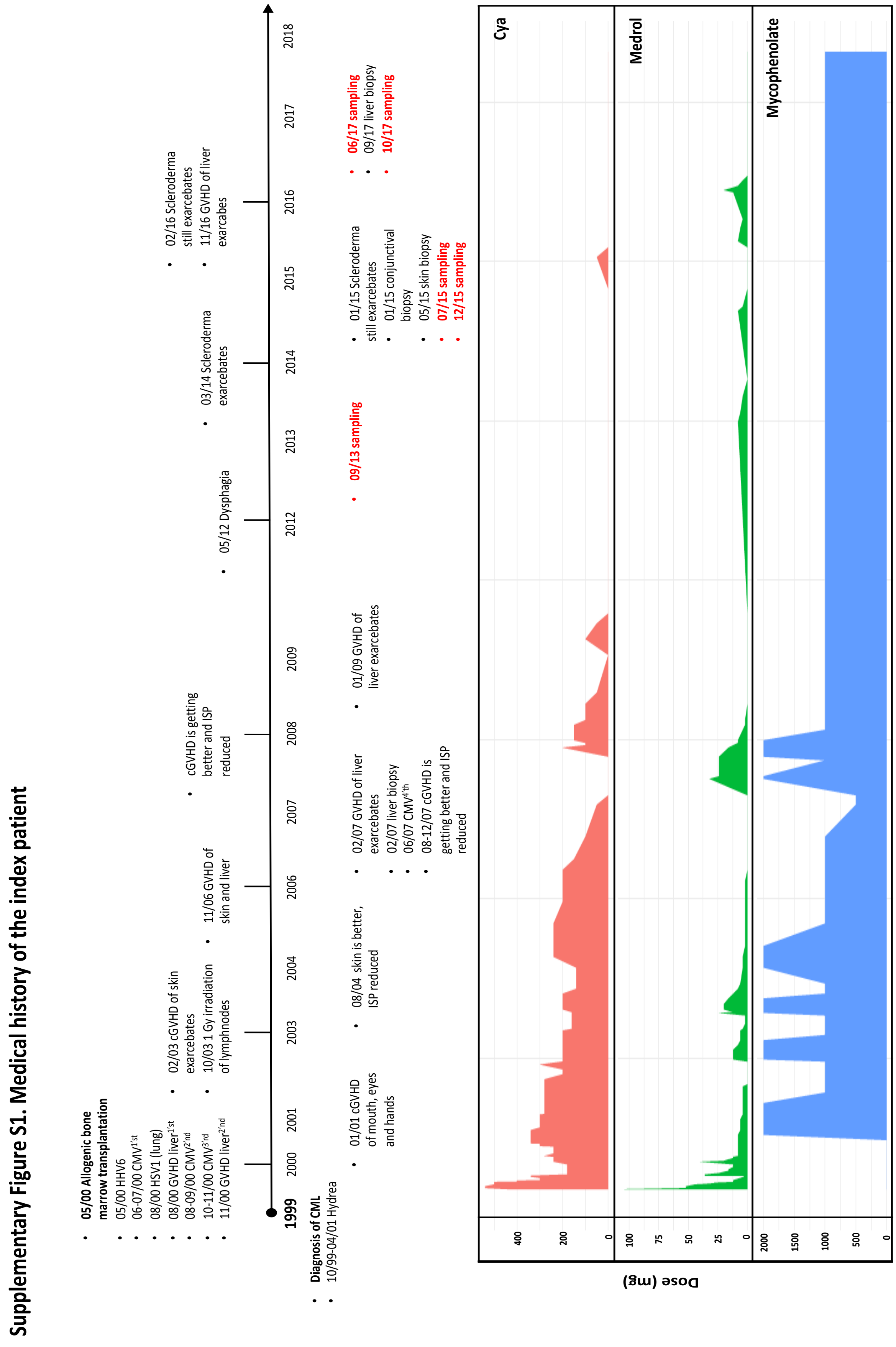

Supplementary Figure 2. Flow cytometry analysis of the index patient

a Lymphocyte population in peripheral blood (Index patient)

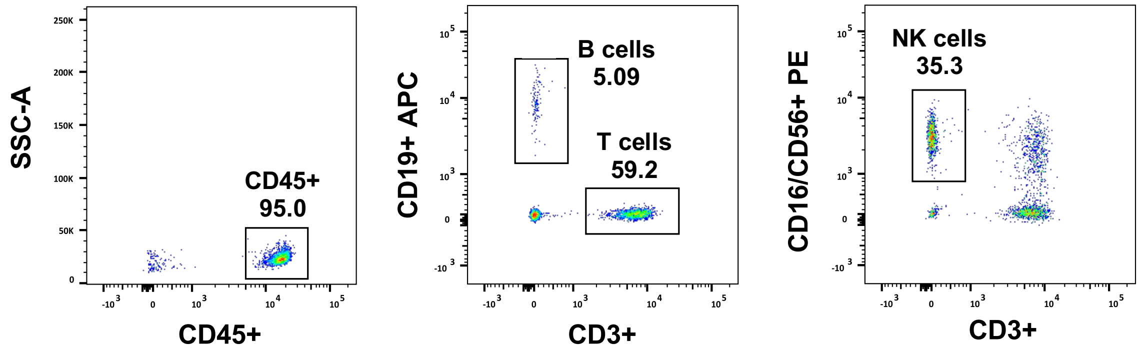

b Gating strategy for flow-assisted cell sorting (Index patient)

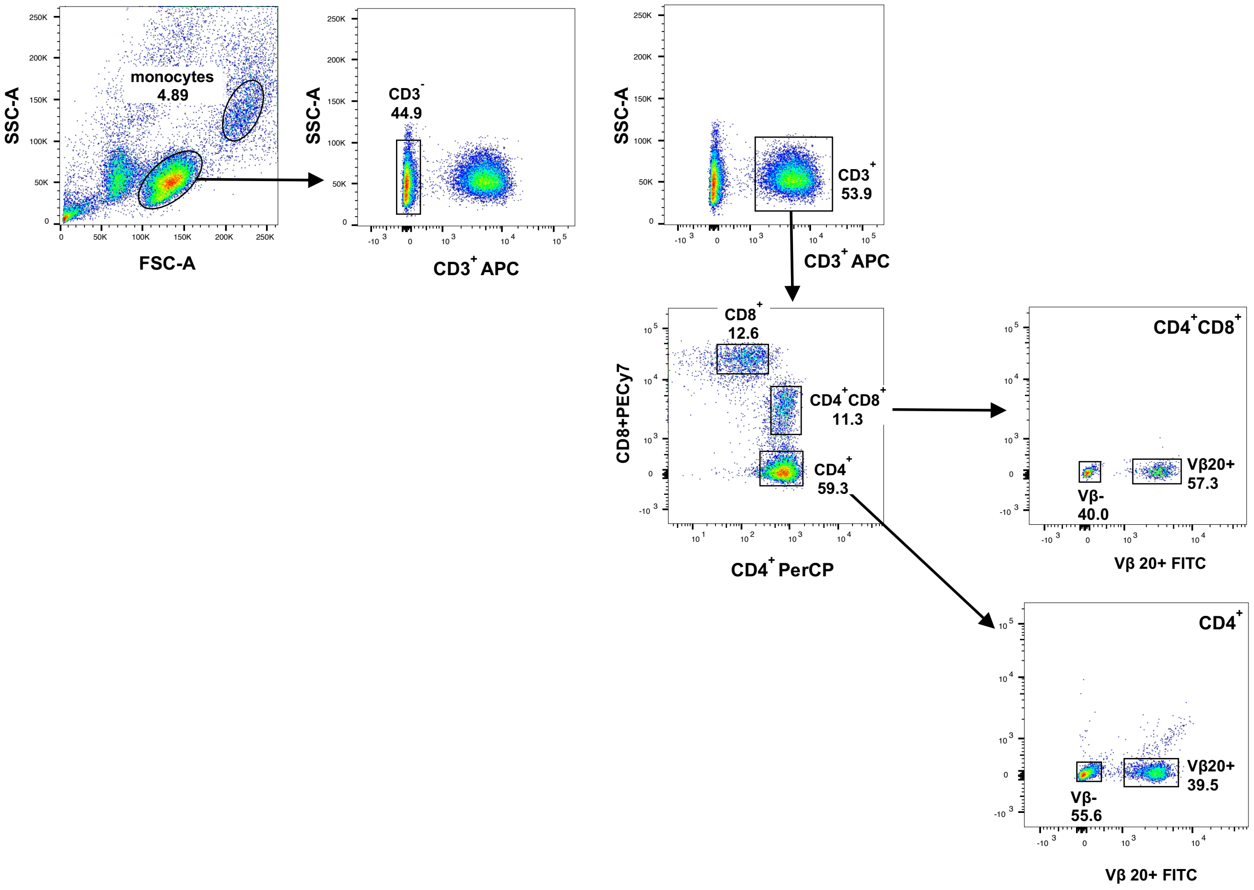

c CD4[+] T cells and CD8[+] T cells stained with TCR Vβ antibodies (Index patient)

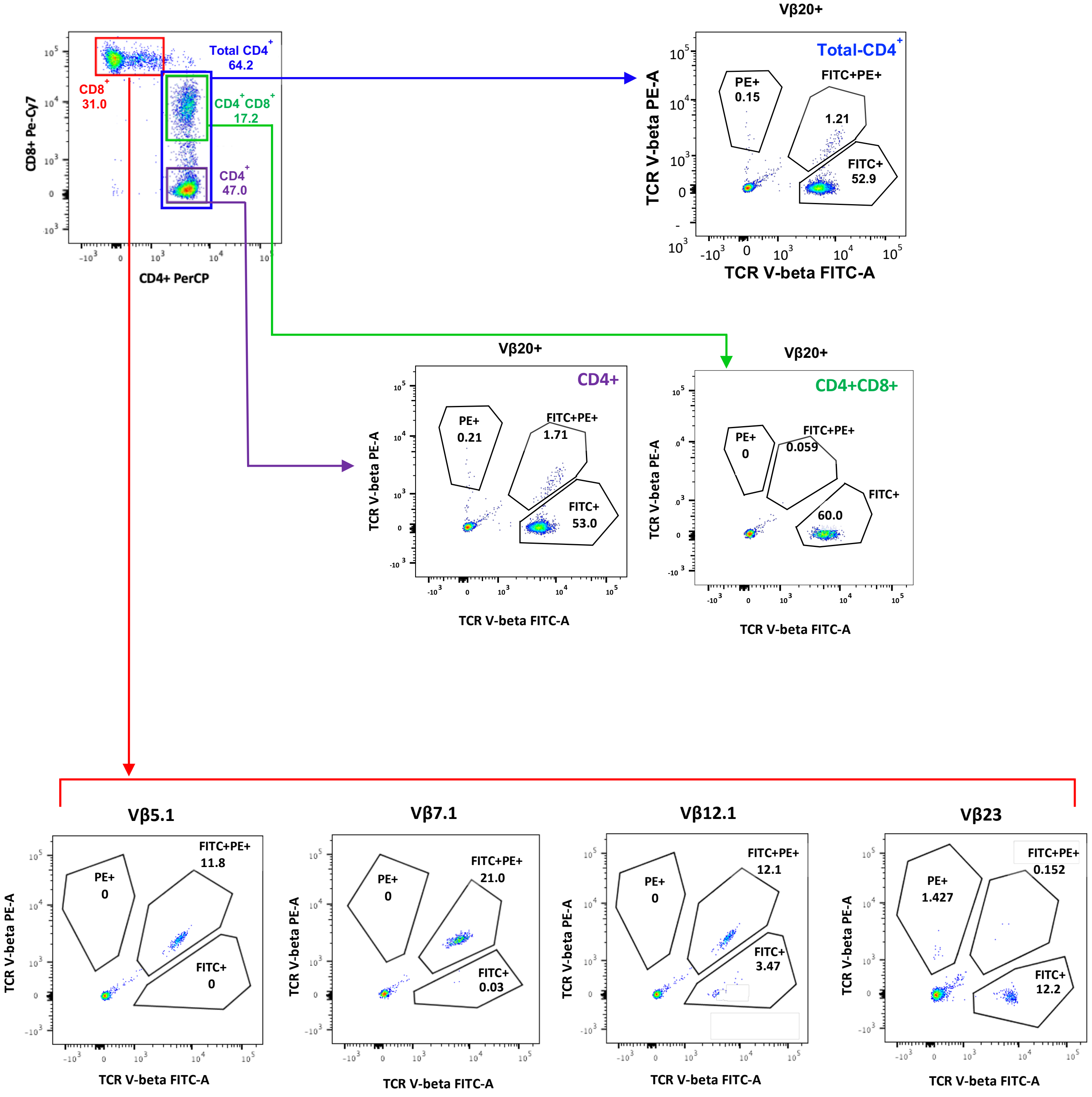

d Gating strategies used for flow cytometry analysis.

Figure 1 a

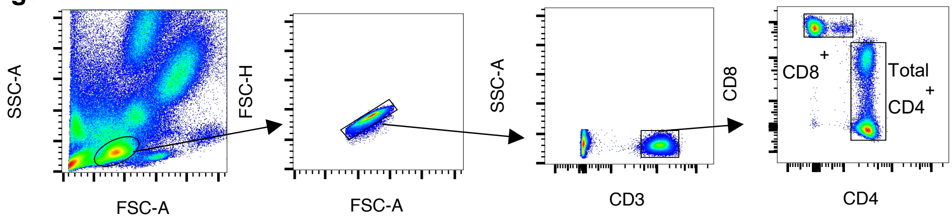

Figure 1 e

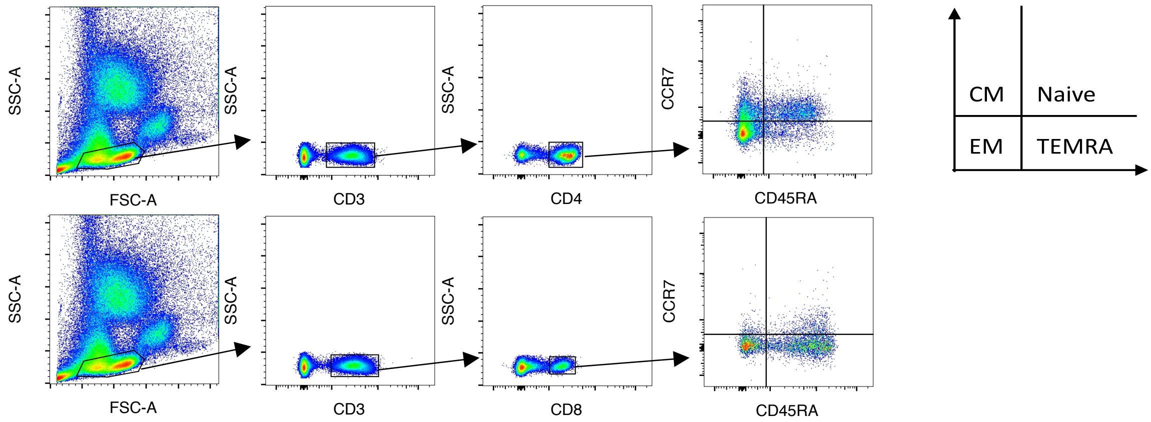

Figure 1 f

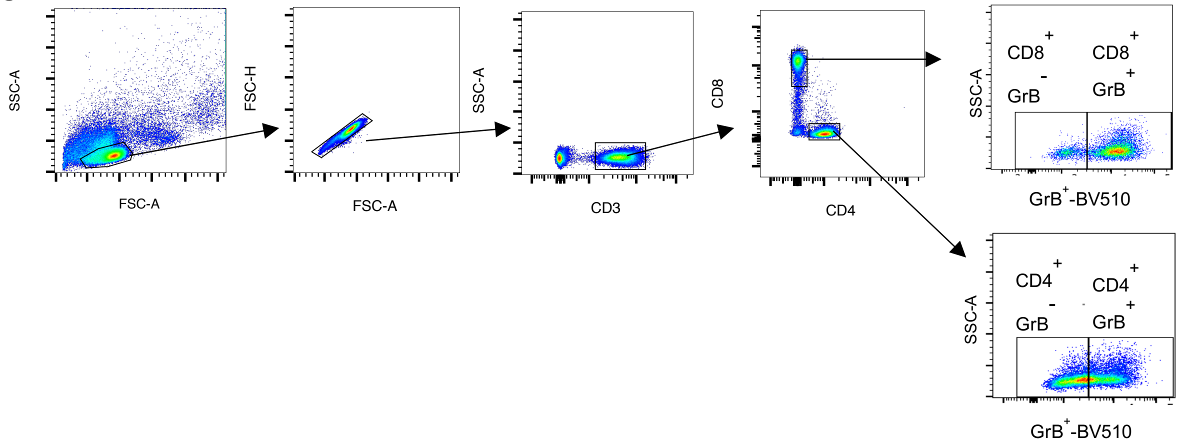

Supplementary Figure 2. Flow cytometry analysis of the index patient

a Basic lymphocyte immunophenotyping was performed to identify T ($CD3^+$), natural killer (NK) ($CD3^-CD16^+CD56^+$), and B ($CD3^-CD19^+$) cells by flow cytometry.

b Gating strategy for flow-based cell sorting. Monocytes were gated based on morphological features using SSC-A and FSC-A plots. $CD3^-$ and $CD3^+$ cells were sorted from lymphocytes based on antibody staining, and $CD3^+$ cells were separated to $CD4^+$, $CD4^+CD8^+$ and $CD8^+$ fractions. Finally, TCR Vβ20+ and Vβ20- cells were sorted.

c Vβ20 clone constituted 53% of pure $CD4^+$ T cells and 60% of $CD4^+CD8^+$ T cells. Vβ5.1, Vβ7.1, Vβ12.1, and Vβ23 clone constituted 11.8%, 21%, 12,1%, and 12.2% of $CD8^+$ T cells respectively.

d Gating strategies used for cell sorting. Gating strategy to sort $CD4^+$ and $CD8^+$ T cells from PBMCs presented on Fig. 1a. Gating strategy to sort effector memory (EM) cells , terminally differentiated effector memory (TEMRA) cells, and central memory (CM) cells from both $CD4^+$ and $CD8^+$ T cells which are presented on Fig. 1e. Gating strategy to sort $CD4^+$Granzyme B^+ cells and $CD8^+$Granzyme B^+ cells from PBMCs presented on Fig. 1f.

Supplementary Figure 3. Variant allele frequencies of *TLR2* mutations in cGvHD patients' and healthy controls' CD4⁺ and CD8⁺ T cells.

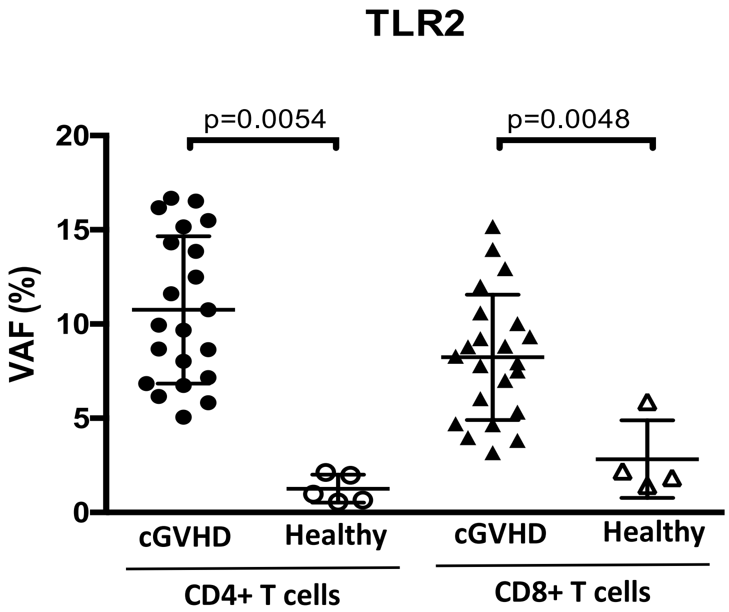

Supplementary Figure 3. Variant allele frequencies of *TLR2* mutations in cGvHD patients' and healthy controls' CD4⁺ and CD8⁺ T cells.

TLR2 mutation analyzed by amplicon sequencing in cGvHD patients (CD4⁺ T, n=21 and CD8⁺ T, n=22) and healthy controls (CD4⁺ T, n=5 and CD8⁺ T, n=4). Mononuclear cells (MNCs) were separated from whole blood with Ficoll-Paque PLUS (GE Healthcare, UK) followed by magnetic beads sorting of both CD4⁺ and CD8⁺ T cells. The purity of sorted fractions was evaluated by flow cytometry (FACSVerse) and confirmed to be >98%.

The analysis indicated 10-fold higher variant allele frequency in cGvHD patients compared to healthy controls. Unpaired t-test was performed with Welch's correction to calculate p value using Graphpad Software (San Diego, USA). Error bar present Mean ± SD. Source data are provided as a Source Data file.

Supplementary Figure 4. Protein expression and immunoprecipitation assay in HEK293 expressing *mTOR* wildtype and *mTOR P2229R* mutant in standard culture condition

a

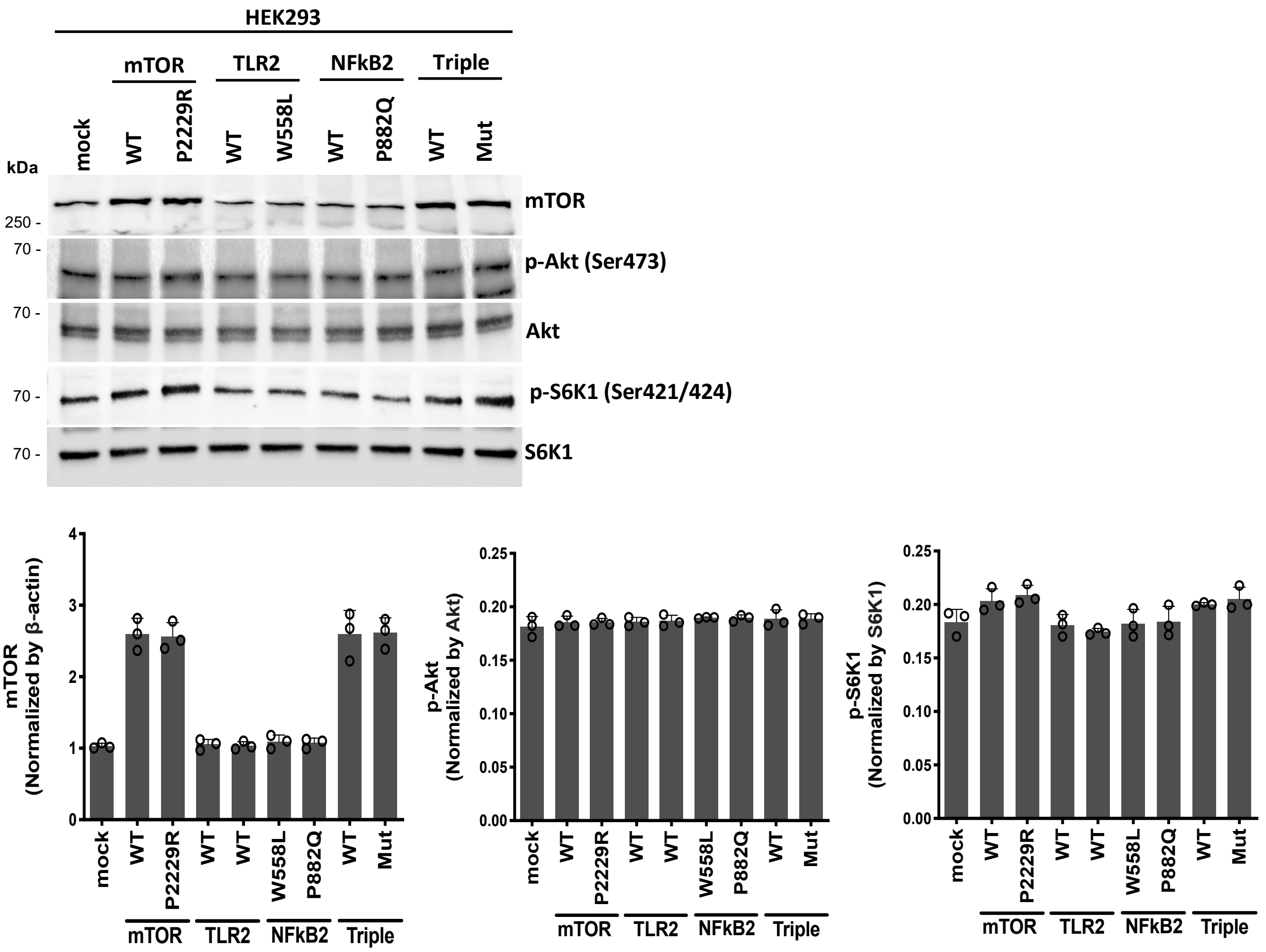

b

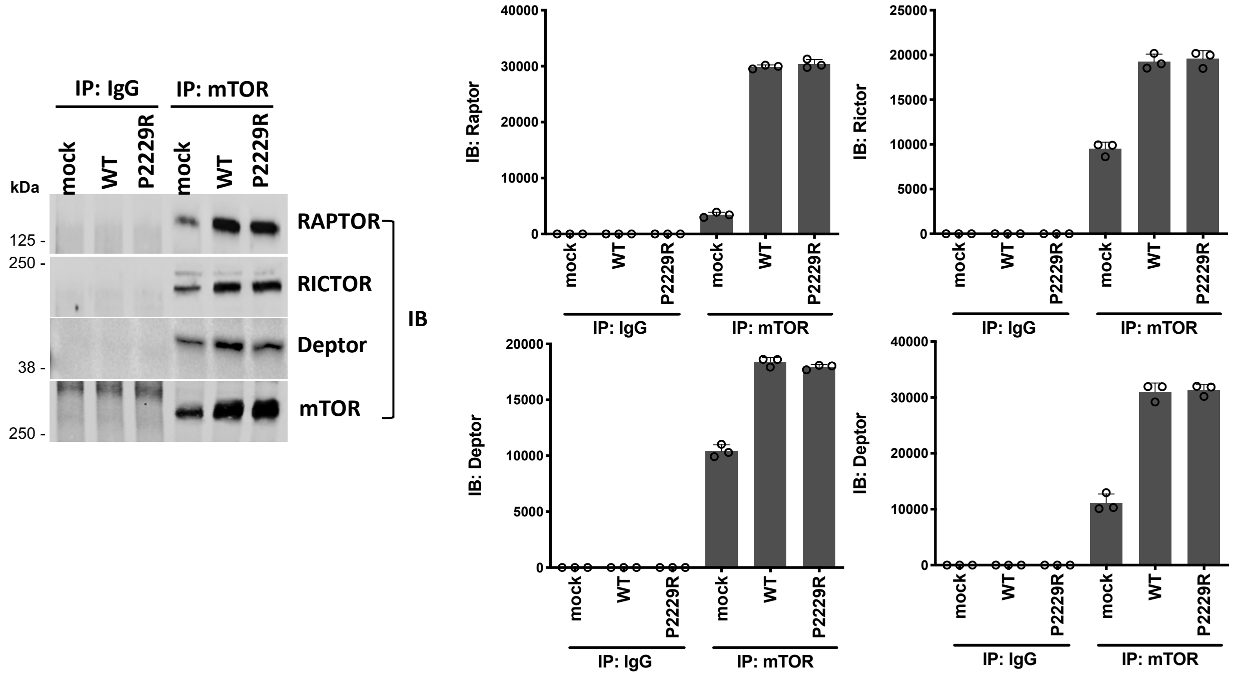

Supplementary Figure 4. Protein expression and immunoprecipitation assay in HEK293 expressing *mTOR* wildtype and *mTOR P2229R* mutant in standard culture condition. a Phosphorylation of Akt and S6K1 in 10% serum condition. Protein levels were quantified using ImageJ. **b** Co-Immunoprecipitation (Co-IP) of HEK293 stably expressing *mTOR WT* and *P2229R* in 10% serum condition. mTOR was immunoprecipitated (IP) with anti-mTOR antibody (1:50 dilution) and immunoblotted (IB) for RAPTOR, RICTOR, and DEPTOR antibodies. P values are derived from Welch's correction ($mTOR^{WT}$ *vs* $mTOR^{P2229R}$). Error bar present Mean ± SD (n=3 per group). Source data are provided as a Source Data file.

Supplementary Figure 5. Quantitative presentation of the protein expression level

a

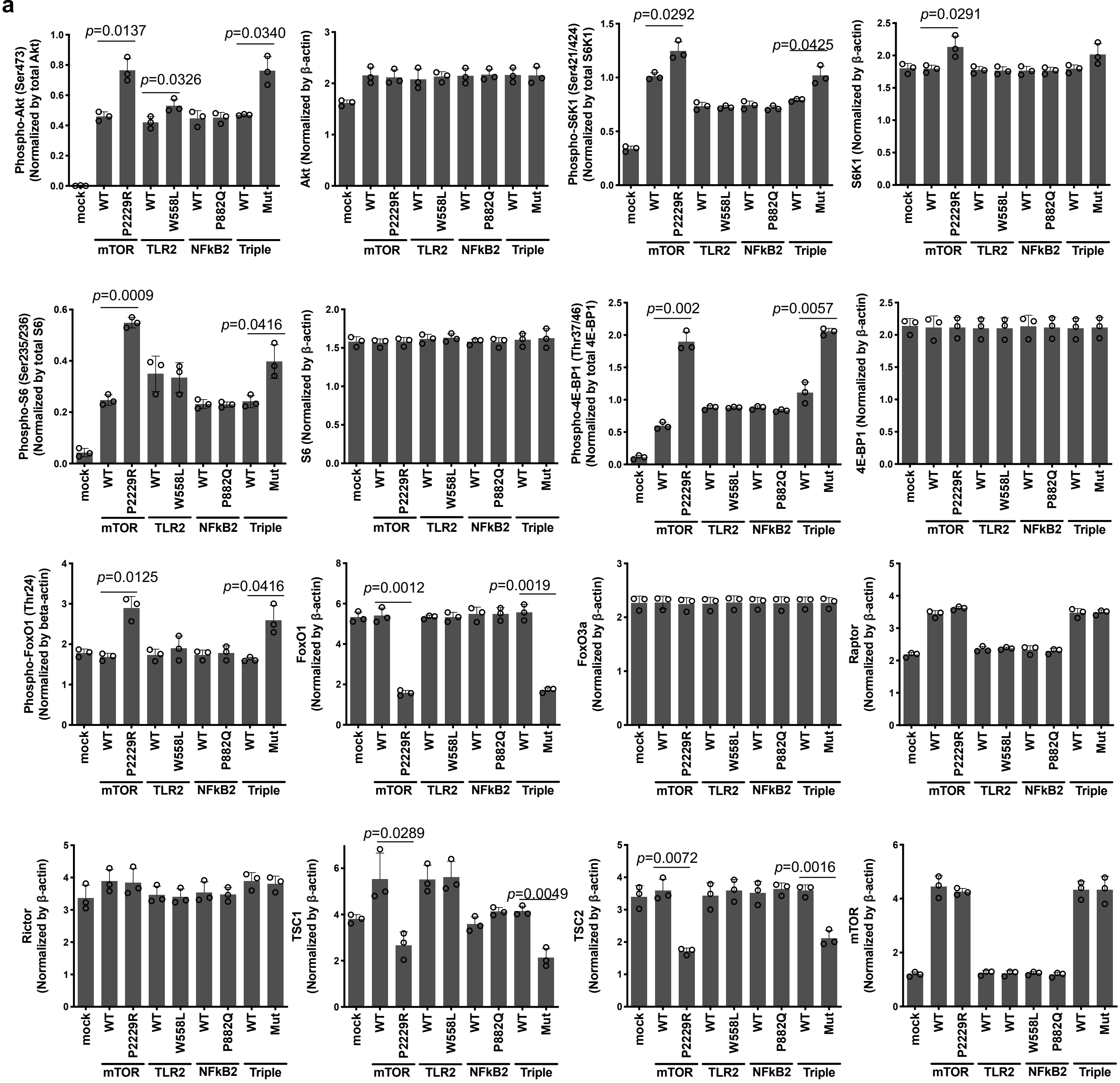

b

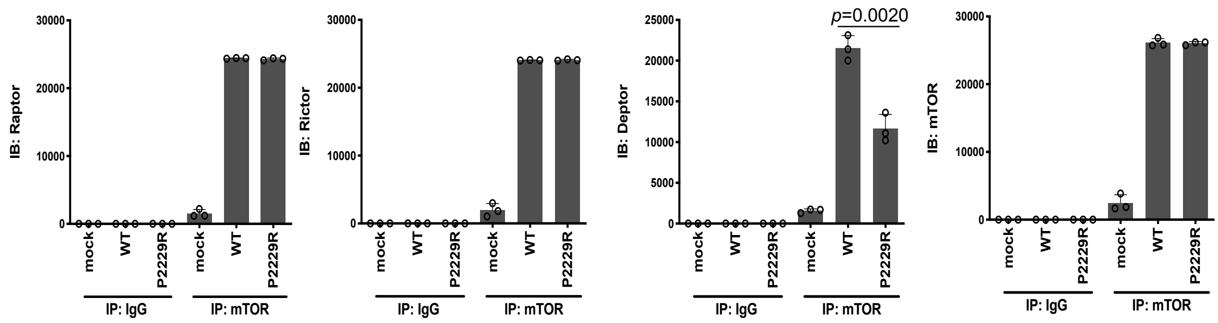

Supplementary Figure 5. Quantitative presentation of the protein expression level presented in Figure 3d and Figure 3e.

Quantitative presentation of the western blot assays using ImageJ software (version 2.0.0). **a** Protein levels were first normalized with β-actin level. Phosphorylation levels were normalized by dividing the intensities of phosphorylated protein level by total protein level. P values are derived from unpaired t-test with Welch's correction (*$mTOR^{WT}$ vs $mTOR^{P2229R}$, $TLR2^{WT}$ vs $TLR2^{W558L}$, $NFkB2^{WT}$* vs *$NFkB2^{P882Q}$, $Triple^{WT}$ vs $Triple^{MUT}$*). Source data are provided as a Source Data file. **b** Protein levels were quantified using ImageJ. P values are derived from Welch's correction (*$mTOR^{WT}$ vs $mTOR^{P2229R}$*). Error bar present Mean ± SD (n=3 per group)

Supplementary Figure 6. Functional analysis of *NFkB2* mutant and *TLR2* mutant in HEK293 cells

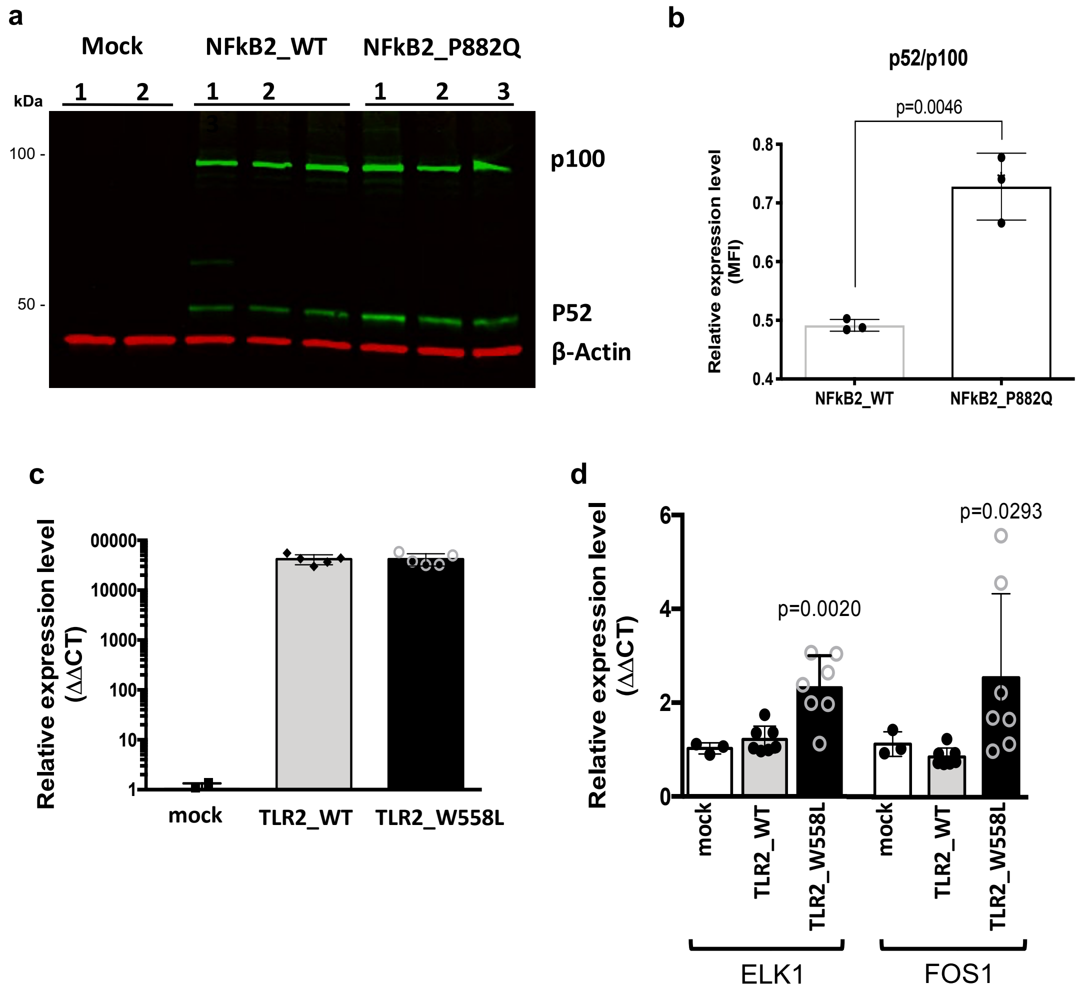

Supplementary Figure 6. Functional analysis of *NFkB2* mutant and *TLR2* mutant in HEK293 cells

a The alteration of P52 and P100 expression with *NFkB2_WT* and *NFkB2_P882Q* mutant was verified by western blot. Data is representative of three independent experiments. Source data are provided as a Source Data file.

b Stably expressing *NFkB2 P882Q* increased expression of P52. Mean fluorescence intensity was measured by ImageJ software (*NFkB2_WT*: n =3, *NFkB2_P882Q*: n=3). Error bar present Mean ± SD. P values are derived from unpaired t-test with Welch's correction (*NFkB2_WT* vs *NFkB2_P882Q*). Source data are provided as a Source Data file.

c The effect of *TLR2 W558L* mutant on the downstream gene expression. RT-qPCR was performed for *TLR2* with empty vector (mock, n=2), *TLR2_WT* (n=5) and *TLR2_W558L* (n=5).

d *ELK1* and *FOS1* genes using stably expressed empty vector (mock, n=3), *TLR2_WT* (n=7) and *TLR2_W558L* (n=7) in HEK293 cells was identified by RT-qPCR. *TLR2_W558L* increased expression level of downstream target genes, *ELK1* and *FOS1*. Unpaired t-test was performed with Welchi's correction to calculate p-value (*TLR2_WT* vs *TLR2_W558L*) using Graphpad Software (San Diego, USA). Error bar present Mean ± SD.

Supplementary Figure 7. Single cell analysis of sorted $CD4^{+}$ T cells from 2015 and 2017 timepoints (Index patient)

a Gene expression fold change within CD4+ T cell clusters between 2015 and 2017

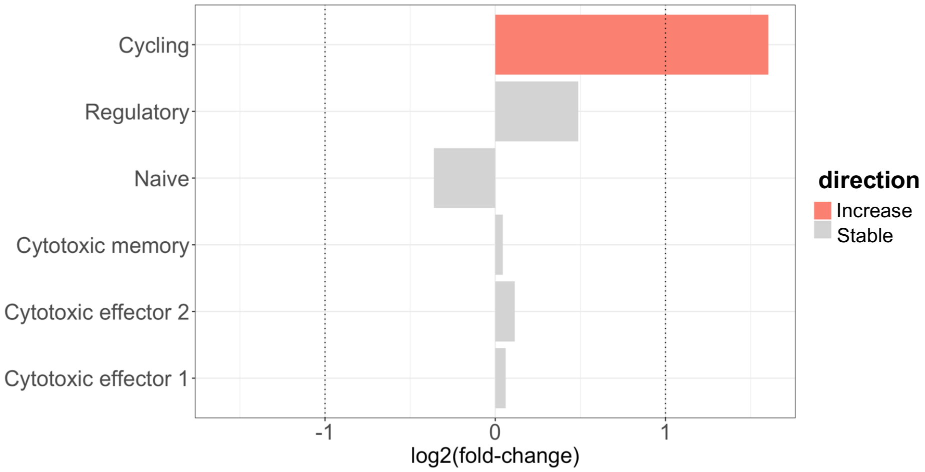

b Dimensional reduction analyses from 2015 and 2017

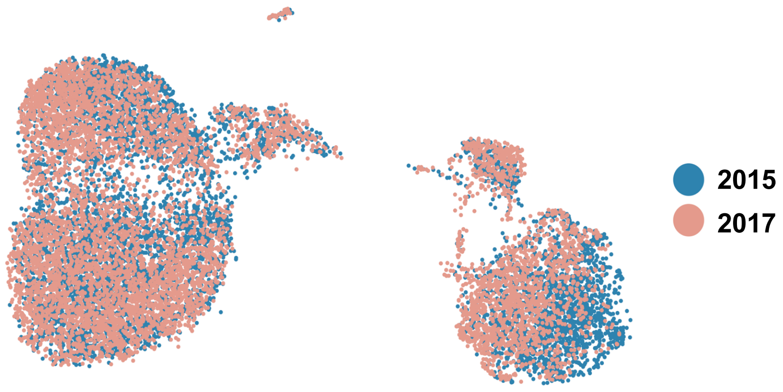

c Number of cells from clusters

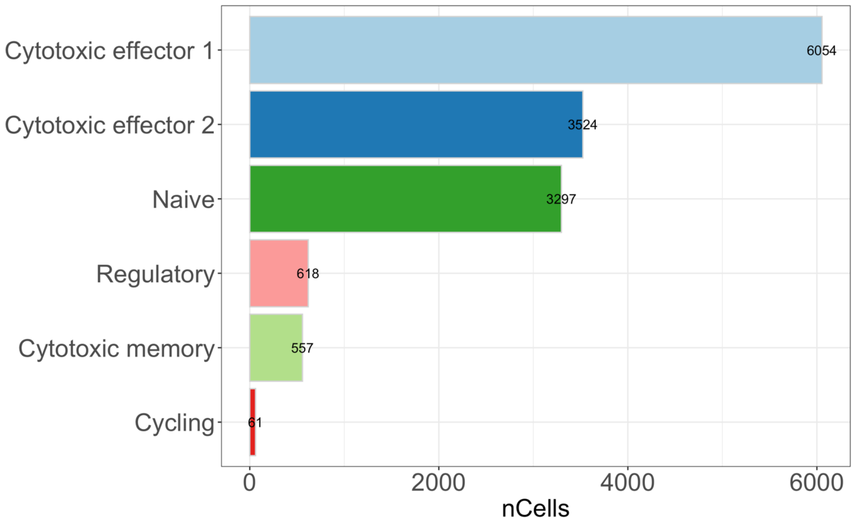

Supplementary Figure 7. Single cell analysis of sorted CD4⁺ T cells from 2015 and 2017 timepoints (Index patient)

Simultaneous single-cell RNA and paired TCRαβ sequencing was performed on two time points (2015 and 2017) for the index patient's $CD4^+$ T lymphocytes from peripheral blood. In the data analysis phase, samples were pooled together to examine possible differences between the time points.

a Comparison of the number of cells in different clusters between two timepoints. Only the abundance of cycling cell population showed over two-fold-change between the two timepoints (marked with red color).

b Two-dimension UMAP-projection of clustered $CD4^+$ T cells pooled from two timepoints from peripheral blood. Cells from 2015 are highlighted with blue color and from 2017 with red color. Cells from two different samples merged well with each other.

c Number of cells belonging to 6 different $CD4^+$ T cell clusters

The read counts of scRNA-seq data are deposited in the ArrayExpress database at EMBL-EBI (www.ebi.ac.uk/arrayexpress) under the accession number E-MTAB-8911.

Supplementary Figure 8. Granzyme B positive (GrB+) T cell populations and Th-1 type cytokine production in GvHD patients and healthy controls.

a Granzyme B$^+$ population in CD4$^+$ T cells

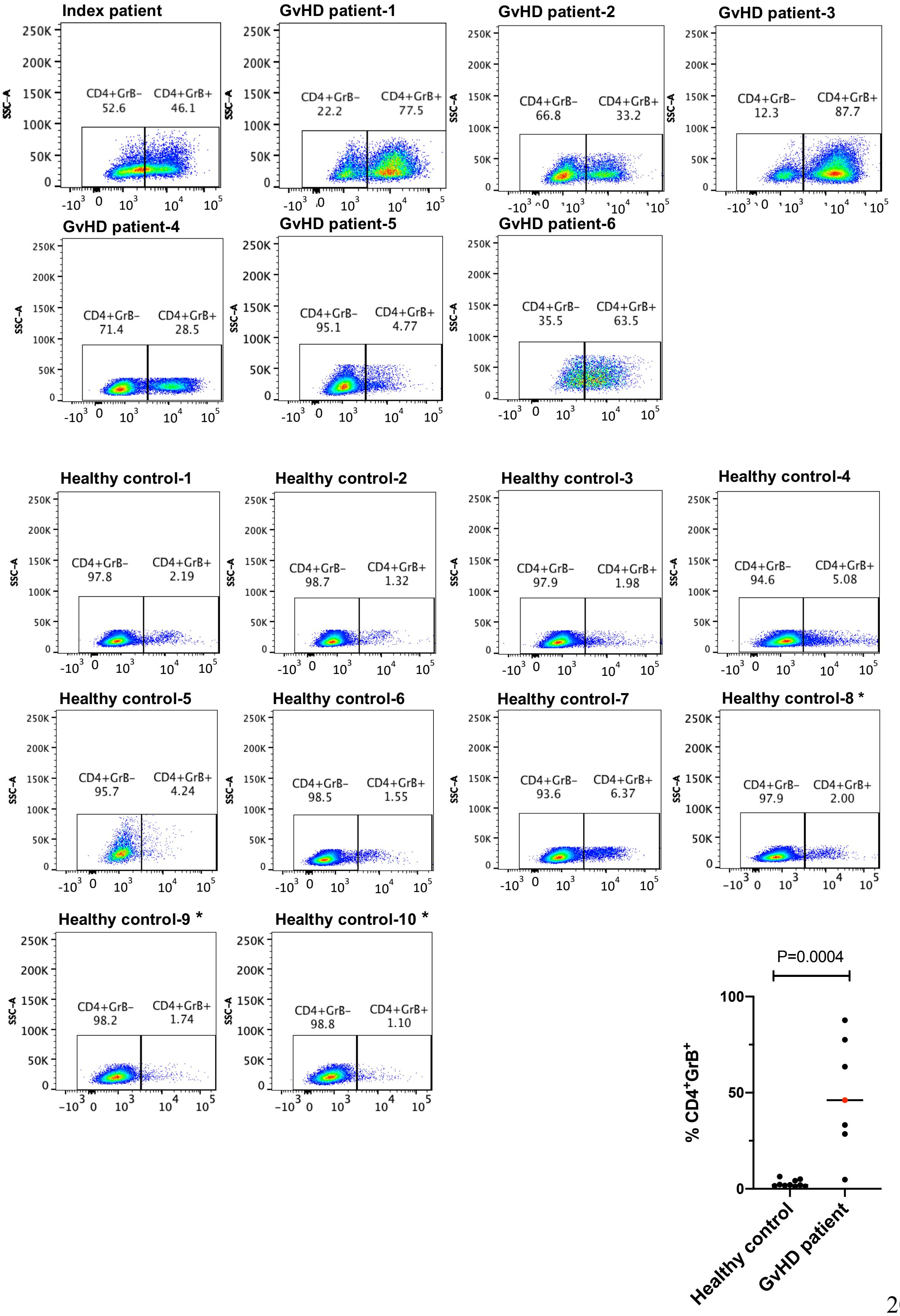

b Granzyme B^+ population in $CD8^+$ T cells

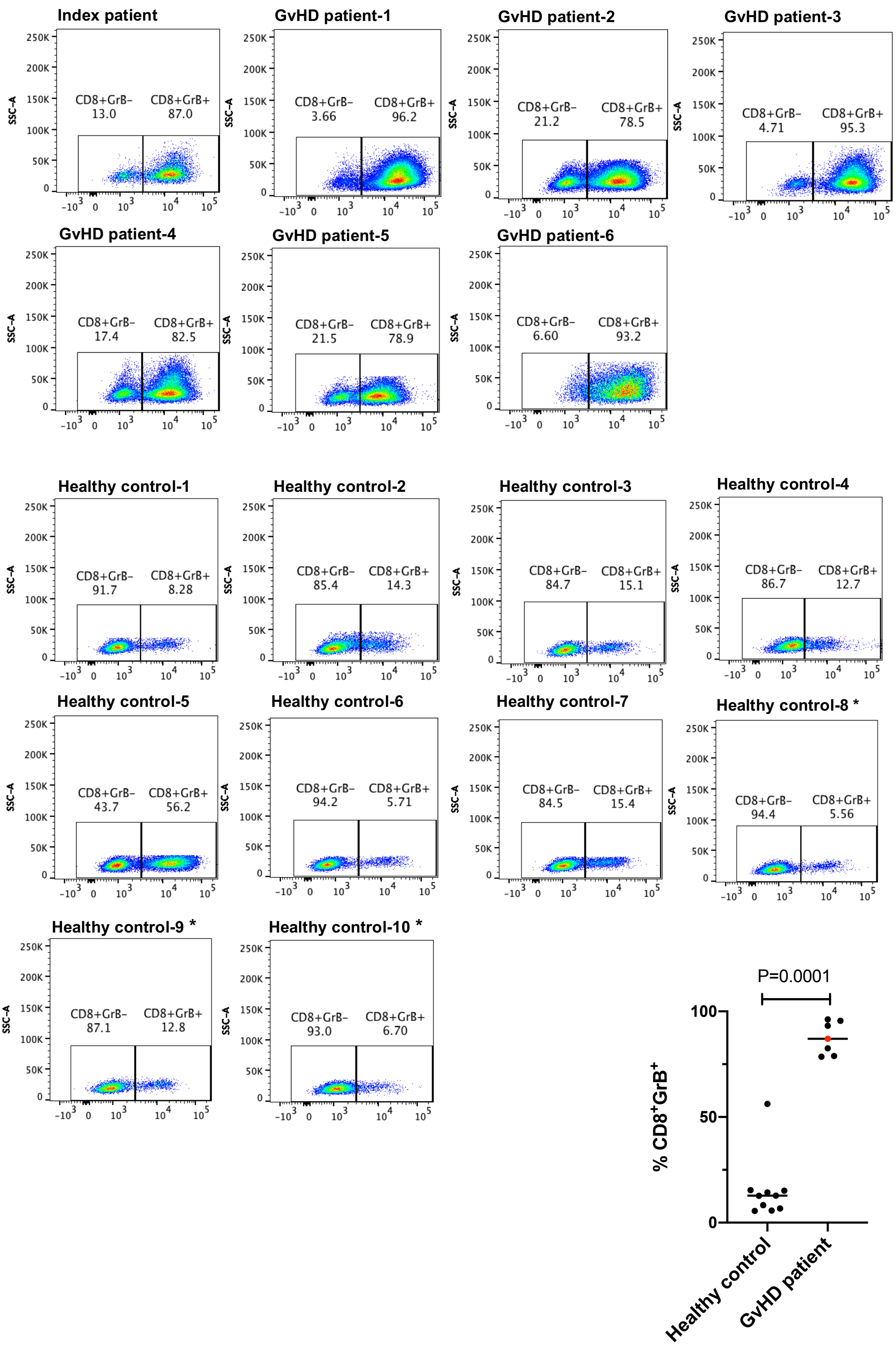

c TNF-α + INF-γ production in CD4^{+}Granzyme B^{+}

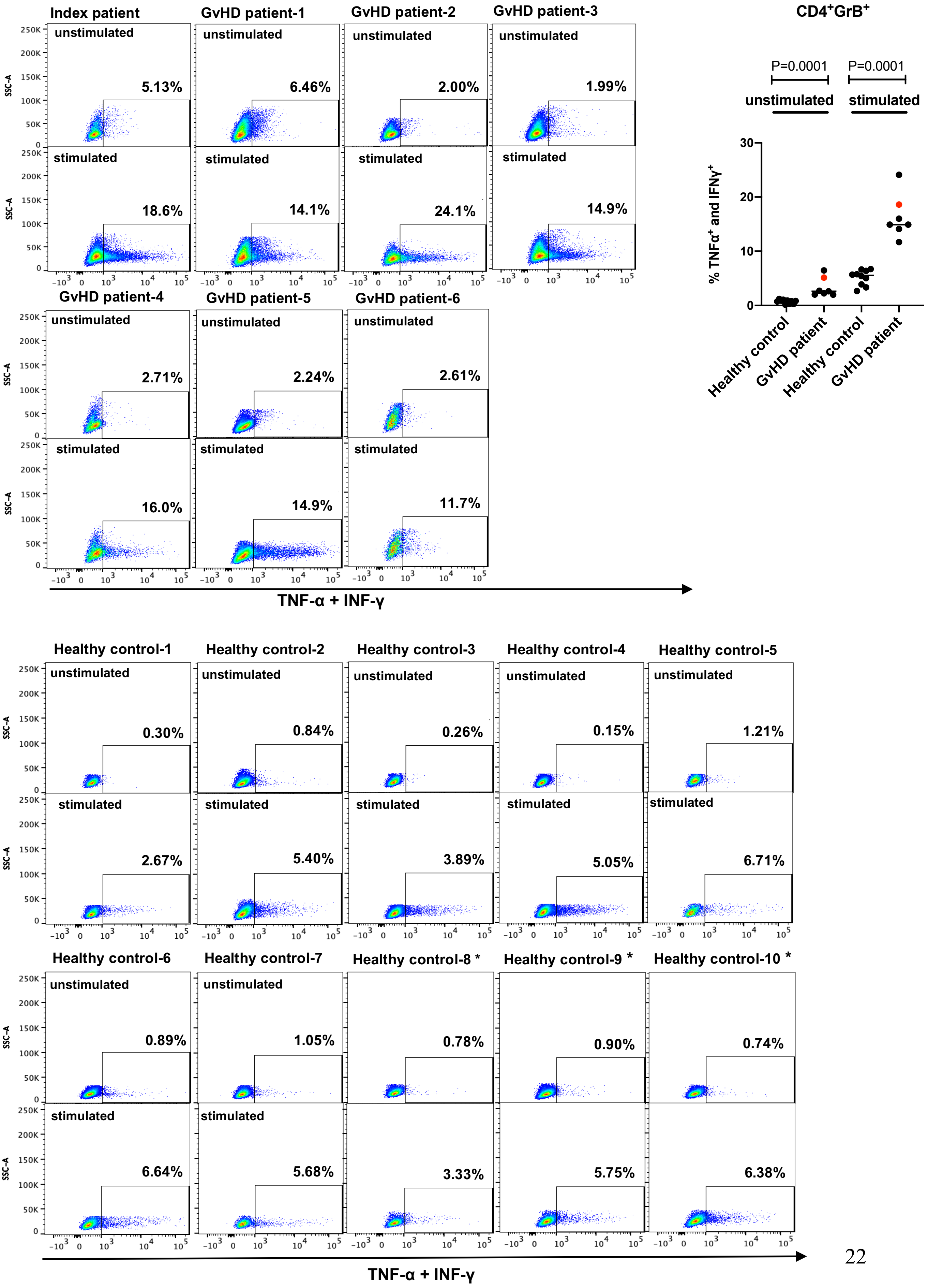

d TNF-α + INF-γ production in CD8^{+}Granzyme B^{+}

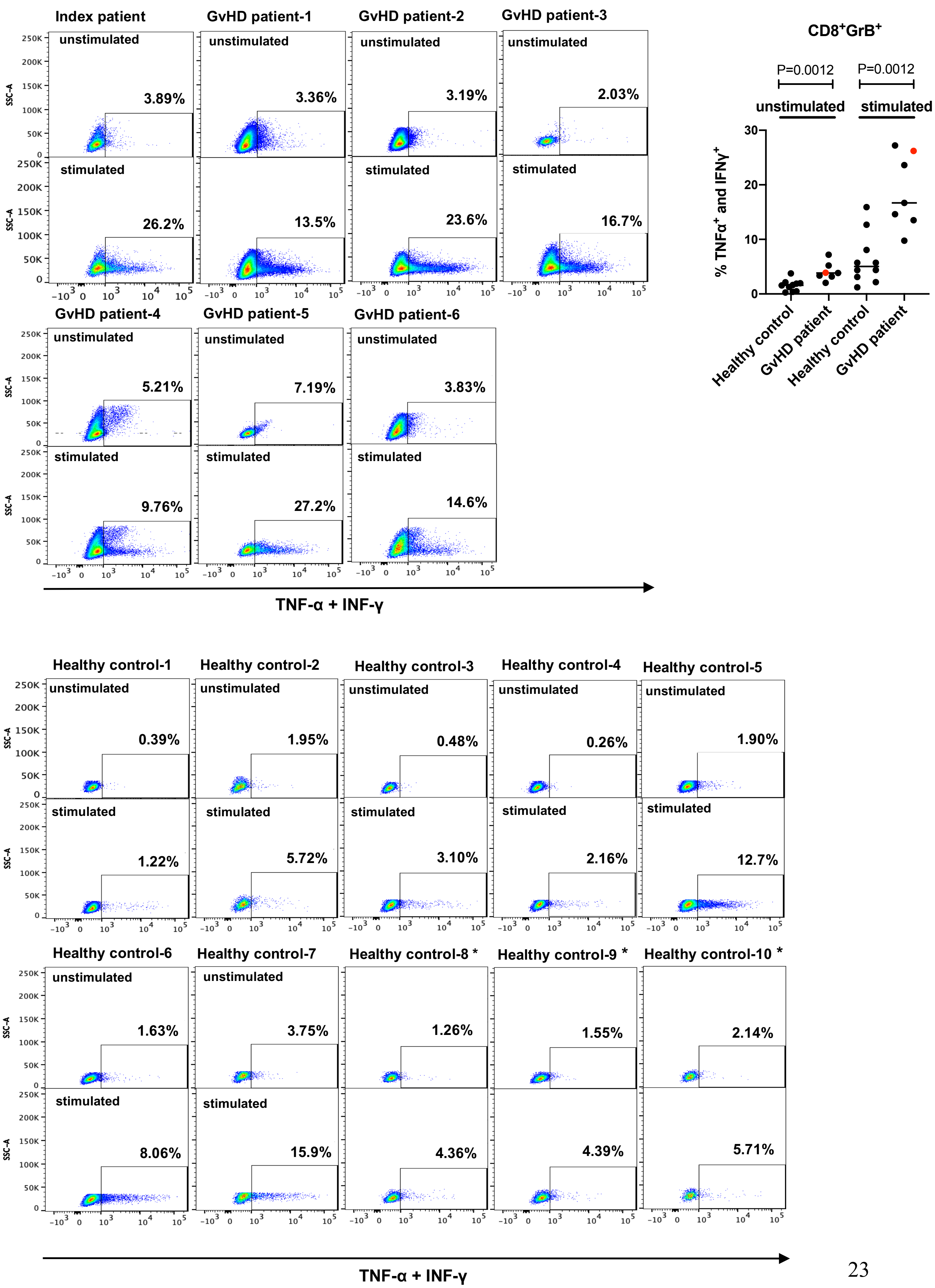

Supplementary Figure 8. Granzyme B positive (GrB+) T cell populations and Th-1 type cytokine production in GvHD patients and healthy controls.

PBMCs of GvHD patients and healthy controls were stained with anti-CD45, -CD3, -CD4, and -CD8 (surface markers), and then granzyme B (GrB) was stained with fixation and permeabilization. Stained cells were analyzed using FACSVerse (BD). The relative proportion of **(a)** granzyme B positive (GrB^+) $CD4^+$ T cells (GrB^+CD4^+) and **(b)** GrB^+CD8^+ T cells from cGvHD patients (n=7) and healthy controls (n=10). For T cell stimulation, anti-CD3 (0.33ug/mL), -CD28 (1μg/mL), and -CD49 (1μg/mL) were added to the media with GolgiSTOP (protein transport inhibitor containing 99.61% ethanol w/w and 0.26% monensin w/w) followed by 16 h incubation at 37°C in RPMI1640. Th1-type cytokine (TNF-α and IFN-γ) production by **(c)** GrB^+CD4^+ T cells and **(d)** GrB^+CD8^+ T cells were measured from non-stimulated T cells and stimulated T cells by flow cytometry. The summary data are shown with each point representing an individual assessed in each group. Red dot indicates the index patient's sample. P values are derived from nonparametric Mann-Whitney t-test using GraphPad Prism (Ver8.3.0). Index patient's sample taken on July 2015 was used. Healthy controls used in the cytotoxicity assay in supplementary figure 9b-d.

Supplementary Figure 9. Real-time Cell cytotoxicity assay of CD4⁺ T cells and CD8⁺ T cells from index patient and health donors

a

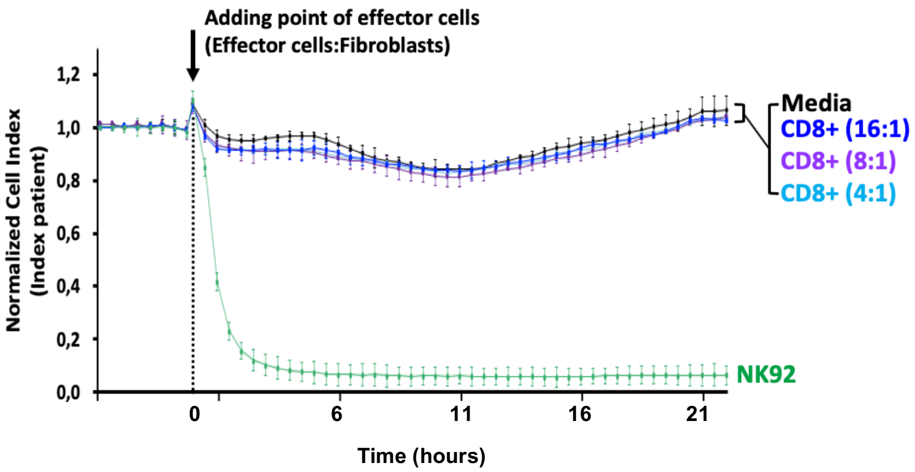

b

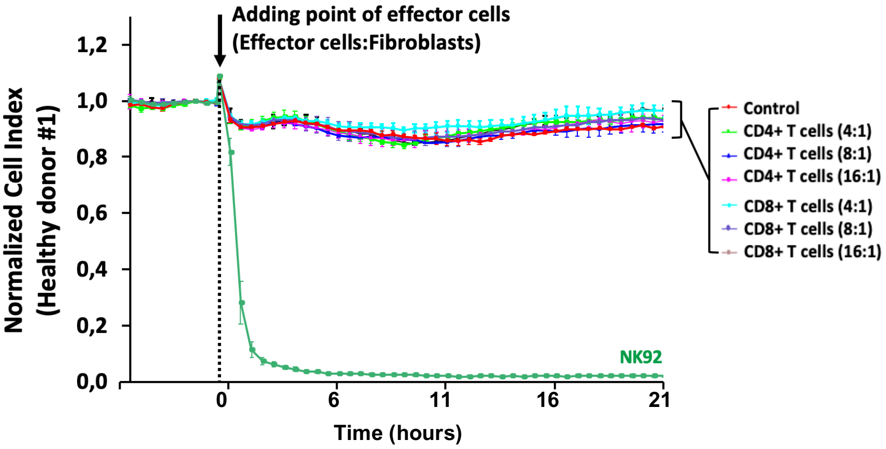

c

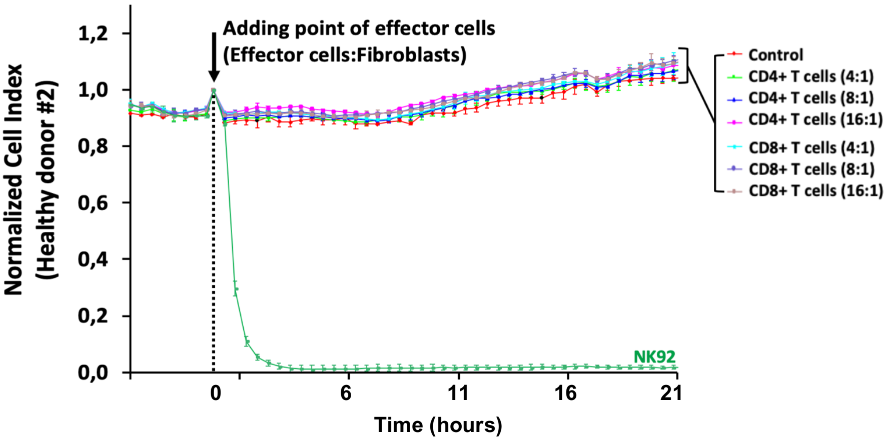

d

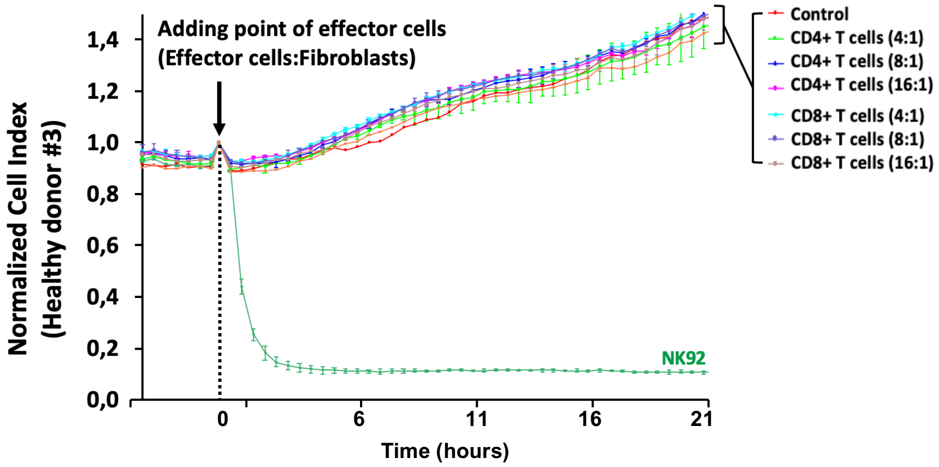

Supplementary Figure 9. Real-time Cell cytotoxicity assay of CD4$^+$ T cells and CD8$^+$ T cells from index patient and health donors

Primary fibroblasts (8 x 10^3 cells) from skin biopsy were cultured for 24 hours. Either CD4+ T cells or CD8+ T cells or NK92 cells were added with different ratio to patient's **(a)** or three healthy donors' own fibroblasts **(b-d)** followed by co-culture up to 24 hours. The measured impedance was expressed as Cell Index with the normalization (n=2). Arrow indicates the adding point of CD4+, CD8+ T cells and NK92 (0 h). Dots represent mean values and error bars indicate range (n=2 for all conditions, technical duplicates). Source data are provided as a Source Data file.

Supplementary Figure 10. Real-time Cell cytotoxicity analysis of CD4$^+$ T cells with ganetespib and sirolimus from index patient.

a

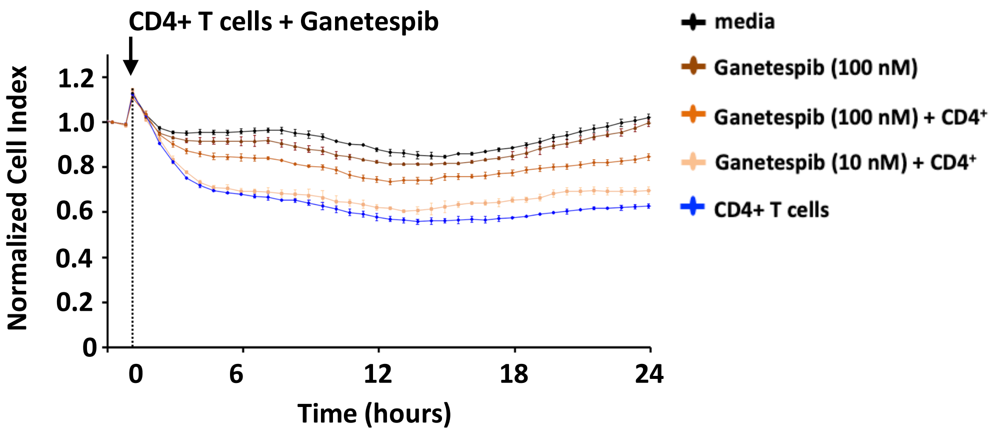

b

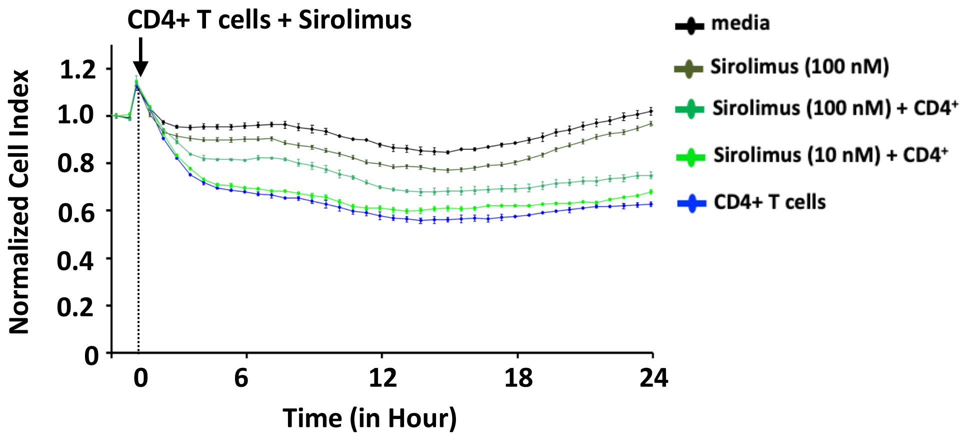

Supplementary Figure 10. Real-time Cell cytotoxicity analysis of CD4$^+$ T cells with ganetespib and sirolimus from index patient.

Primary fibroblasts (8 x 10^3 cells) from skin biopsies were cultured for 24 hours. CD4$^+$ T cells (8:1=CD4$^+$ T cells:fibroblasts) were added with **(a)** ganetespib (HSP90 inhibitor, 10nM and 100nM) **(b)** and sirolimus (mTOR inhibitor, 10nM and 100nM) followed by co-culture up to 24 hours. The measured impedance was expressed as Cell Index with the normalization (n=2). Arrow indicates the adding point of CD4$^+$ T cells and the inhibitors (0 h). Dots represent mean values and error bars indicate range (n=2 for all conditions, technical duplicates). Source data are provided as a Source Data file.

Supplementary Figure 11. Relative protein expression presented in Fig. 6f-g.

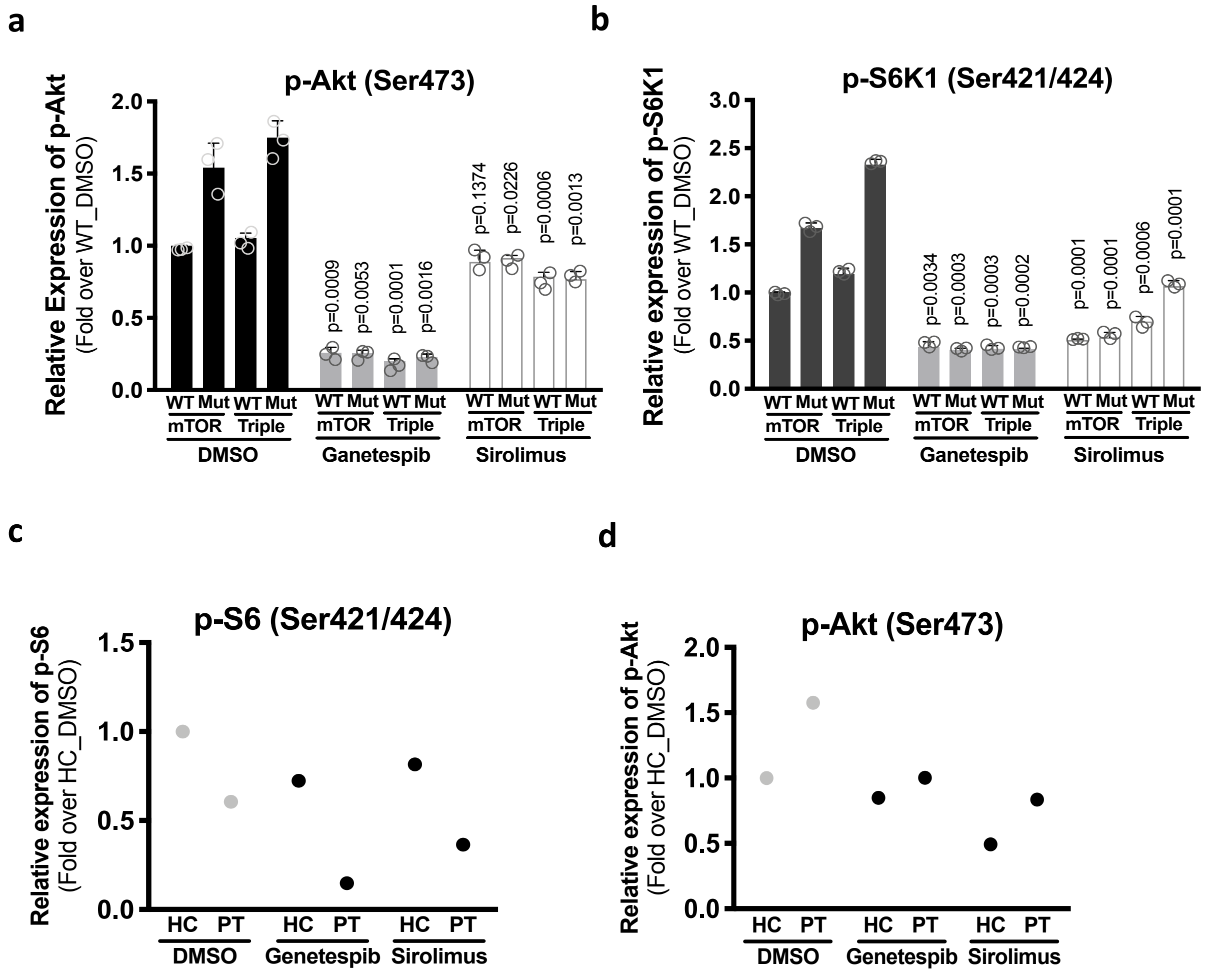

Supplementary Figure 11. Relative expression levels of **(a)** phospho-Akt and **(b)** phospho-S6K1 were estimated by measuring each band intensity using ImageJ software (Rasband, W.S., ImageJ, U. S. National Institutes of Health, Bethesda, Maryland, USA, https://imagej.nih.gov/ij/, 1997-2016). Error bar present Mean ± SD (n=3 per group). P values are derived from unpaired t-test with Welch's correction (p value on each bar indicates statistical significance between DMSO and Ganetespib/Sirolimus). **(c), (d)** Relative expression levels of phospho-S6 and phospho-Akt were estimated by measuring band intensity with ImageJ software. Source data are provided as a Source Data file.

Supplementary Table 1. Immunogene panel and whole exome sequencing results for the index patient

(a) CD8+ T-cells purified in 2013 (immunogene panel)

Chr	Position	Ref	Var	Gene	Mutation type	Codon change	Exon	Amino acid change	CD4_ Ref reads[1]	CD4_ Alt reads[2]	CD4_ Var_ Freq (%)	CD8_ Ref reads[3]	CD8_ Alt reads[4]	CD8_ var_ Freq (%)	Somatic p-value*
17	80274141	GCAGGGA<br>GGGCAGA<br>GGCTGCT<br>GGCGGGT	G	CD7	CODON CHANGE PLUS DELETION	Gacccgccag<br>Cagcctctgcc<br>ctccctgcg/<br>gcg	3	DPPAASA<br>LPA172A	19	0	0	8	7	46.67	0.0011962

(b) CD4+ T-cells purified in 2015 (whole exome sequencing)

Chr	Position	Ref	Var	Gene	Mutation type	Codon change	Exon	Amino acid change	CD8_ Ref reads[1]	CD8_ Alt reads[2]	CD8_ Var_ Freq (%)	CD4_ Ref reads[3]	CD4_ Alt reads[4]	CD4_ var_ Freq (%)	Somatic p-value*
22	29885598	AAG<br>GAA<br>G	A	NEFH	CODON_ DELETION	aaggaagag<br>/aag	4	KEE657K	141	0	0	114	21	15.56	1.2713E-07
22	30198134	A	T	ASCC2	MISSENSE	Tcc/Acc	14	S473T	48	0	0	28	13	31.71	0.000012532
11	45671492	C	T	CHST1	MISSENSE	Gtg/Atg	4	V328M	76	0	0	48	11	18.64	0.000062539
17	8160230	C	A	PFAS	MISSENSE	Cac/Aac	9	H342N	48	0	0	26	10	27.78	0.000092062
1	236906279	G	T	ACTN2	MISSENSE	gaG/gaT	11	E397D	91	0	0	72	11	13.25	0.000201
4	100573255	C	T	RP11-<br>766F14.2.<br>1	MISSENSE	Gcc/Acc	1	A851T	83	0	0	56	9	13.85	0.00043634
17	66986988	T	C	ABCA9	MISSENSE	gAc/gGc	29	D1276G	44	0	0	18	7	28	0.00044555
12	6181540	G	A	VWF	MISSENSE	Cgc/Tgc	9	R356C	56	0	0	45	10	18.18	0.00056748
10	104162075	C	A	NFKB2	MISSENSE	cCa/cAa	23	P882Q	113	0	0	75	8	9.64	0.0008439
3	119886213	C	T	GPR156	MISSENSE	cGg/cAg	10	R704Q	58	0	0	37	8	17.78	0.00090659
22	30927925	C	T	SEC14L6	MISSENSE	Gat/Aat	6	D162N	66	0	0	43	8	15.69	0.00093377
9	34834390	C	A	AL589645.<br>1	MISSENSE	Gtg/Ttg	4	V367L	138	0	0	113	9	7.38	0.00093765
1	11182160	G	C	MTOR	MISSENSE	cCt/cGt	48	P2229R	55	0	0	23	6	20.69	0.0011686
4	154625732	G	T	TLR2	MISSENSE	tGg/tTg	1	W558L	63	0	0	54	7	11.48	0.0057957
2	44428602	TGGA<br>AAAT<br>CAG	T	PPM1B	FRAME_ SHIFT	-/-	2	-89	88	0	0	63	6	8.7	0.0063483
17	72926604	G	T	OTOP2	MISSENSE	Ggc/Tgc	6	G292C	85	0	0	65	6	8.45	0.0078858
7	141401764	G	A	KIAA1147	MISSENSE	Ccc/Tcc	1	P64S	16	0	0	5	4	44.44	0.0099605

(c) CD8+ T-cells purified in 2015 (whole exome sequencing)

Chr	Position	Ref	Var	Gene	Mutation type	Codon change	Exon	Amino acid change	CD4_ Ref reads [1]	CD4_ Alt reads[2]	CD4_ Var_ Freq (%)	CD8_ Ref reads [3]	CD8_ Alt reads[4]	CD8_ var_ Freq (%)	Somatic p-value*
22	26879946	GGAGGC GGCGCC CCGGGG GAGA	G	SRRD	DELETION	gaggcgg cgccccgg gggaga/-	1	EAAPR GR31-	10	0	0	6	7	53.85	0.0069996
21	47612645	G	C	AP001468.1	MISSENSE	cCc/cGc	2	P223R	32	1	3.03	30	10	25	0.0085105
12	53069222	CACCTC CGGAGC CGTAGC TGCT	C	KRT1	DELETION	agcagcta cggctccg gaggt/-	9	SSYGS GG557-	8	0	0	13	14	51.85	0.008646

(d) NK cells purified in 2015 (whole exome sequencing)

Chr	Position	Ref	Var	Gene	Mutation type	Codon change	Exon	Amino acid change	Normal_ reads [1]	Normal_ reads [2]	Normal_ var_ Freq (%)	NK_ reads [3]	NK_ reads [4]	NK_ var_ Freq (%)	Somatic p-value*
12	21997770	C	T	ABCC9	STOP_GAINED	tGg/tAg	25	W1059*	70	0	0	78	32	29.09	1.7846E-08
18	28935163	G	A	DSG1	MISSENSE	Ggc/Agc	15	G1002S	60	0	0	69	14	16.87	0.00029757
1	231830034	T	A	DISC1	MISSENSE	cTg/cAg	2	L177Q	81	0	0	72	10	12.2	0.00077705
1	75606686	G	A	LHX8	STOP_GAINED	tGg/tAg	5	W95*	37	0	0	34	10	22.73	0.0013209
2	233839411	T	C	NGEF	MISSENSE	Atc/Gtc	2	I64V	52	0	0	51	10	16.39	0.0014524
1	89449434	T	C	RBMXL1	MISSENSE	Aca/Gca	2	T26A	28	1	3.45	19	10	34.48	0.0027031
6	43266374	C	T	SLC22A7	MISSENSE	aCg/aTg	1	T93M	28	0	0	16	6	27.27	0.0046954

Abbreviations: Chr. chromosome; ref. reference base; var. variant base; freq. frequency
1 Sequencing reads supporting reference allele in normal sample
2 Sequencing reads supporting variant allele in normal sample
3 Sequencing reads supporting reference allele in tumor sample
4 Sequencing reads supporting variant allele in tumor sample
* Somatic p-value for somatic/loss of heterozygosity events

Supplementary Table 2. Somatic *NFkB2*, *TLR2* and *mTOR* mutations in different cellular fractions (Supplementary Figure S2b) from the index patient validated by amplicon sequencing

DNAs	Gene	Chr	Position	Ref	Var	Amino acid change	Total_Depth	Call_Depth	Ref_Calls	Var_Calls	VAF (%)	Freq_ Ratio
CD3neg	NFKB2	10	104162075	C	A	P882Q	49669	49669	49640	29	0	0
CD3neg	TLR2	4	154625732	G	T	W558L	102539	102539	102411	128	0	0
CD3neg	MTOR	1	11182160	G	C	P2229R	133746	133746	133628	118	0	0
$CD8^+$	NFKB2	10	104162075	C	A	P882Q	13945	13944	13936	8	0	0
$CD8^+$	TLR2	4	154625732	G	T	W558L	125028	125028	124912	116	0	0
$CD8^+$	MTOR	1	11182160	G	C	P2229R	156055	156055	155995	60	0	0
$CD4^+$Vb.20neg	NFKB2	10	104162075	C	A	P882Q	21047	21047	21024	23	0	0
$CD4^+$Vb.20neg	TLR2	4	154625732	G	T	W558L	149991	149991	149151	840	0.5	0.98095
$CD4^+$Vb.20neg	MTOR	1	11182160	G	C	P2229R	184213	184213	183230	983	0	0
$CD4^+CD8^+$ Vb.20neg	NFKB2	10	104162075	C	A	P882Q	66813	66812	66301	511	0.7	0.98661
$CD4^+CD8^+$ Vb.20neg	TLR2	4	154625732	G	T	W558L	124985	124985	123059	1926	1.5	1.00066
$CD4^+CD8^+$ Vb.20neg	MTOR	1	11182160	G	C	P2229R	170803	170803	167577	3226	1.9	0.99731
Monocytes	NFKB2	10	104162075	C	A	P882Q	40654	40653	40618	35	0	0
Monocytes	TLR2	4	154625732	G	T	W558L	147684	147684	146326	1358	0.9	0.99539
Monocytes	MTOR	1	11182160	G	C	P2229R	172128	172128	169802	2326	1.3	0.99164

The purity of sorted fractions was evaluated by flow cytometry (FACS AriaII) and confirmed to be >98%

Abbreviations: Chr. chromosome; ref. reference base; var. variant base; VAF. variant allele frequency; Freq. frequency; Freq_Ratio. base quality frequency ratio

Supplementary Table 3. Somatic *TLR2* mutation in $CD4^+$ T cells validated by amplicon sequencing

	Patient	DNAs	Gene	Chr	position	ref	var	Amino acid change	Total_Depth	Call_Depth	Ref_Calls	Var_Calls	VAF (%)	Freq_ Ratio
1	Index	$CD4^+$ T	TLR2	4	154625732	G	T	W558L	543370	543370	453452	89918	16.5	1.00623
1	Index	$CD4^+$Vb.20	TLR2	4	154625732	G	T	W558L	160135	160135	103031	57104	35.5	1.01047
1	Index	$CD4^+CD8^+$ Vb.20	TLR2	4	154625732	G	T	W558L	123431	123431	89029	34402	27.8	1.01117
1	Index (2013)	$CD4^+$ T	TLR2	4	154625732	G	T	W558L	703	703	588	115	16.1	0.95745
1	Index (2017)	$CD4^+$ T	TLR2	4	154625732	G	T	W558L	974	974	840	134	13.3	1.01498
1	Index (2019)	$CD4^+$ T	TLR2	4	154625732	G	T	W558L	974	974	803	171	17.5	1.01014
2	GVHD	$CD4^+$ T	TLR2	4	154625732	G	T	W558L	39775	39775	34242	5533	13.9	1.01118
3	GVHD	$CD4^+$ T	TLR2	4	154625732	G	T	W558L	31285	31285	26791	4494	14.3	1.01125
4	GVHD	$CD4^+$ T	TLR2	4	154625732	G	T	W558L	17162	17162	15779	1383	8.0	1.01096
5	GVHD	$CD4^+$ T	TLR2	4	154625732	G	T	W558L	31044	31044	27152	3892	12.5	1.01145
6	GVHD	$CD4^+$ T	TLR2	4	154625732	G	T	W558L	15876	15876	15067	809	5.1	1.00573
7	GVHD	$CD4^+$ T	TLR2	4	154625732	G	T	W558L	28823	28823	26310	2513	8.7	1.00554
8	GVHD	$CD4^+$ T	TLR2	4	154625732	G	T	W558L	16932	16932	15291	1641	9.7	1.0061
9	GVHD	$CD4^+$ T	TLR2	4	154625732	G	T	W558L	19309	19309	17983	1326	6.8	1.0079
10	GVHD	$CD4^+$ T	TLR2	4	154625732	G	T	W558L	28042	28042	25247	2795	9.9	1.00633
11	GVHD	$CD4^+$ T	TLR2	4	154625732	G	T	W558L	33628	33628	28176	5452	16.2	1.00513
12	GVHD	$CD4^+$ T	TLR2	4	154625732	G	T	W558L	34034	34034	31716	2318	6.7	1.00415
13	GVHD	$CD4^+$ T	TLR2	4	154625732	G	T	W558L	27185	27185	24024	3161	11.6	1.01008
14	GVHD	$CD4^+$ T	TLR2	4	154625732	G	T	W558L	8934	8934	8290	644	7.2	1.00475
15	GVHD	$CD4^+$ T	TLR2	4	154625732	G	T	W558L	25657	25657	21757	3900	15.2	1.01141
16	GVHD	$CD4^+$ T	TLR2	4	154625732	G	T	W558L	24821	24821	23284	1537	6.2	1.00601
17	GVHD	$CD4^+$ T	TLR2	4	154625732	G	T	W558L	32618	32617	29085	3532	10.8	1.01143
18	GVHD	$CD4^+$ T	TLR2	4	154625732	G	T	W558L	20425	20425	19223	1202	5.8	1.01546
19	GVHD	$CD4^+$ T	TLR2	4	154625732	G	T	W558L	24791	24791	20940	3851	15.5	1.00852
20	GVHD	$CD4^+$ T	TLR2	4	154625732	G	T	W558L	32973	32973	30111	2862	8.6	1.00359
21	GVHD	$CD4^+$ T	TLR2	4	154625732	G	T	W558L	456	456	379	77	16.7	1.02202
1	control	$CD4^+$ T	TLR2	4	154625732	G	T	W558L	32297	32297	32066	231	0.7	0.97297
2	control	$CD4^+$ T	TLR2	4	154625732	G	T	W558L	17599	17599	17490	109	0.6	0.97691
3	control	$CD4^+$ T	TLR2	4	154625732	G	T	W558L	28586	28586	28296	290	1.0	0.98678
4	control	$CD4^+$ T	TLR2	4	154625732	G	T	W558L	35632	35632	34855	777	2.1	1.00516
5	control	$CD4^+$ T	TLR2	4	154625732	G	T	W558L	22900	22900	22428	472	1.9	0.99899

Abbreviations: Chr. chromosome; ref. reference base; var. variant base; VAF. variant allele frequency; Freq. frequency; Freq_Ratio. base quality frequency ratio

Supplementary Table 4. Somatic *TLR2* mutation in CD8$^+$ T cells validated by amplicon sequencing

	Patient	DNAs	Gene	Chr	position	ref	var	Amino acid change	Total_Depth	Call_Depth	Ref_Calls	Var_Calls	VAF (%)	Freq_ Ratio
1	Index	CD8$^+$T	TLR2	4	154625732	G	T	W558L	999903	999903	952631	47272	4.701	1.00787
2	GVHD	CD8$^+$T	TLR2	4	154625732	G	T	W558L	12699	12699	11570	1129	8.827	1.00725
3	GVHD	CD8$^+$T	TLR2	4	154625732	G	T	W558L	18488	18488	16762	1726	9.233	1.0104
4	GVHD	CD8$^+$T	TLR2	4	154625732	G	T	W558L	40286	40286	34158	6128	15.181	1.01074
5	GVHD	CD8$^+$T	TLR2	4	154625732	G	T	W558L	54276	54276	51710	2566	4.656	1.00365
6	GVHD	CD8$^+$T	TLR2	4	154625732	G	T	W558L	36682	36682	33917	2765	7.51	1.01079
7	GVHD	CD8$^+$T	TLR2	4	154625732	G	T	W558L	15920	15920	14589	1331	8.285	1.00579
8	GVHD	CD8$^+$T	TLR2	4	154625732	G	T	W558L	19640	19640	17092	2548	12.943	1.01113
9	GVHD	CD8$^+$T	TLR2	4	154625732	G	T	W558L	37171	37171	34262	2909	7.791	1.00821
10	GVHD	CD8$^+$T	TLR2	4	154625732	G	T	W558L	24258	24258	21334	2924	12.008	1.00883
11	GVHD	CD8$^+$T	TLR2	4	154625732	G	T	W558L	29187	29187	26608	2579	8.795	1.0008
12	GVHD	CD8$^+$T	TLR2	4	154625732	G	T	W558L	26416	26416	25356	1060	3.967	0.99672
13	GVHD	CD8$^+$T	TLR2	4	154625732	G	T	W558L	20640	20640	19840	800	3.832	1.00522
14	GVHD	CD8$^+$T	TLR2	4	154625732	G	T	W558L	35883	35883	32070	3813	10.596	1.00623
15	GVHD	CD8$^+$T	TLR2	4	154625732	G	T	W558L	42754	42754	36774	5980	13.952	1.01269
16	GVHD	CD8$^+$T	TLR2	4	154625732	G	T	W558L	23141	23141	20973	2168	9.325	1.00794
17	GVHD	CD8$^+$T	TLR2	4	154625732	G	T	W558L	29169	29169	26838	2331	7.95	1.01069
18	GVHD	CD8$^+$T	TLR2	4	154625732	G	T	W558L	17956	17956	16985	971	5.324	1.01146
19	GVHD	CD8$^+$T	TLR2	4	154625732	G	T	W558L	35087	35087	31556	3531	10.032	1.00877
20	GVHD	CD8$^+$T	TLR2	4	154625732	G	T	W558L	20298	20298	19639	659	3.183	1.00346
21	GVHD	CD8$^+$T	TLR2	4	154625732	G	T	W558L	1704	1704	1598	106	6.045	0.99851
22	GVHD	CD8$^+$T	TLR2	4	154625732	G	T	W558L	6416	6416	5960	456	7.014	1.01526
1	control	CD8$^+$T	TLR2	4	154625732	G	T	W558L	10509	10509	10354	155	1.418	1.00353
2	control	CD8$^+$T	TLR2	4	154625732	G	T	W558L	17666	17666	17327	339	1.834	0.99182
3	control	CD8$^+$T	TLR2	4	154625732	G	T	W558L	38778	38778	37897	881	2.192	1.01323
4	control	CD8$^+$T	TLR2	4	154625732	G	T	W558L	19211	19211	18069	1142	5.877	1.01361

Abbreviations: Chr. chromosome; ref. reference base; var. variant base; VAF. variant allele frequency; Freq. frequency; Freq_Ratio. base quality frequency ratio

Supplementary Table 5. Gene Sets Enriched Analysis (GSEA)

NAME	GS<br>follow link to MSigDB	SIZE	ES	NES	NOM p-val	FDR q-val	FWER p-val	RANK AT MAX	LEADING EDGE
HALLMARK_TNFA_ SIGNALING_ VIA_NFKB	HALLMARK_TNFA_ SIGNALING_ VIA_NFKB	109	0.61214536	2.5653322	0	0	0	701	tags=45% list=10% signal=49%
HALLMARK_MYC_ TARGETS_V1	HALLMARK_MYC_ TARGETS_V1	146	0.4813359	2.0846016	0	0.00125	0.002	577	tags=26% list=8% signal=28%
HALLMARK_MYC_ TARGETS_V2	HALLMARK_MYC_ TARGETS_V2	30	0.62682414	2.0738354	0	0.001701389	0.004	957	tags=37% list=14% signal=42%
HALLMARK_IL2_ STAT5_SIGNALING	HALLMARK_IL2_ STAT5_SIGNALING	98	0.44755134	1.8531234	0	0.01579182	0.05	560	tags=33% list=8% signal=35%
HALLMARK_ HYPOXIA	HALLMARK_ HYPOXIA	89	0.45142633	1.8455654	0	0.013891303	0.055	410	tags=20% list=6% signal=21%
HALLMARK_UV_ RESPONSE_UP	HALLMARK_UV_ RESPONSE_UP	83	0.40990487	1.6674103	0.00921659	0.039514486	0,12291667	530	tags=19% list=8% signal=21%
HALLMARK_WNT_ BETA_CATENIN_ SIGNALING	HALLMARK_WNT_ BETA_CATENIN_ SIGNALING	20	0.5251126	1.5915891	0.055737704	0.056211945	0,18958333	559	tags=25%l ist=8% signal=27%
HALLMARK_IL6_ JAK_STAT3_ SIGNALING	HALLMARK_IL6_ JAK_STAT3_ SIGNALING	32	0.4547587	1.5182472	0.050167225	0.073746614	0,26805556	560	tags=28% list=8% signal=30%
HALLMARK_P53_ PATHWAY	HALLMARK_P53_ PATHWAY	115	0.32435682	1.4042011	0.029126214	0.11515751	0,40416667	275	tags=13% list=4% signal=13%
HALLMARK_ ESTROGEN_ RESPONSE_LATE	HALLMARK_ ESTROGEN_ RESPONSE_LATE	60	0.35584706	1.3528782	0.07905138	0.13993226	0,49097222	446	tags=20% list=6% signal=21%
HALLMARK_ ESTROGEN_ RESPONSE_ EARLY	HALLMARK_ ESTROGEN_ RESPONSE_ EARLY	68	0.33882925	1.315431	0.07725322	0.1538032	0,53402778	451	tags=21% list=6% signal=22%
HALLMARK_KRAS_ SIGNALING_DN	HALLMARK_KRAS_ SIGNALING_DN	29	0.36606625	1.2103462	0.20560747	0.23421943	0,63958333	687	tags=21% list=10% signal=23%
HALLMARK_ SPERMATOGENESIS	HALLMARK_ SPERMATOGENESIS	36	0.31607693	1.0638554	0.3537415	0.40609884	0,68680556	1061	tags=25% list=15% signal=29%
HALLMARK_UV_ RESPONSE_DN	HALLMARK_UV_ RESPONSE_DN	66	0.26413462	1.0322094	0.4185022	0.42887282	0,69097222	500	tags=14 list=7% signal=15%
HALLMARK_ EDGEHOG_ SIGNALING	HALLMARK_ HEDGEHOG_ SIGNALING	11	0.22904041	0.5976988	0.8742857	0.96099395	0,04166666	205	tags=9% list=3% signal=9%

Supplementary Table 6. Summary of Study Cohorts

a. Patients with cGvHD

**Patient characteristics**	**N**	**%**
**Total**	135	
**Age at sampling (mean. range)**	48	(16-70)
**Patient sex**		
Female	58	43.0
Male	77	57.0
**Donor sex***		
Female	53	39.3
Male	81	60.0
**Sex mismatch***		
No	73	54.1
Yes	61	45.2
**Diagnosis**		
AML. MDS	63	46.7
ALL	17	12.6
NHL	17	12.6
CLL	2	1.5
HL	4	3.0
MPD	15	11.1
MM	11	8.1
Other	6	4.4
**Donor type**		
Sibling	79	58.5
MUD	56	41.5
**Stem Cell Source**		
Peripheral blood	122	90.4
Bone marrow	13	9.6
**HLA match****		
Match	114	84.4
Mismatch	13	9.6
Haplo	7	5.2
**Conditioning***		
MAC	64	47.4
RIC	70	51.9
**Prophylaxis of acute GVHD**		
Calcineurin-based	125	92.6
mTOR-inhibitor based	10	7.4
**Acute GVHD****		
No	66	48.9
Grade <2	17	12.6
Grade >2	50	37.0
**Chronic GvHD***		
Mild	28	20.7
Moderate-Severe	106	78.5
**Delay from tx to sampling**		
< 12 m	67	49.6
12-36 m	53	39.3
> 36 m	15	11.1

b. Patients without cGvHD

Patient characteristics	N	%
**Total**	38	
**Age at sampling (mean. range)**	48	(16-70)
**Patient sex**		
Female	23	60.5
Male	15	39.5
**Donor sex***		
Female	12	31.6
Male	26	68.4
**Sex mismatch***		
No	20	52.6
Yes	18	47.4
**Diagnosis**		
AML. MDS	23	60.5
ALL	6	15.8
NHL	2	5.3
CLL	2	5.3
HL	3	7.9
MPD	1	2.6
MM	0	0
Other	1	2.6
**Donor type**		
Sibling	23	60.5
MUD	15	39.5
**Stem Cell Source**		
Peripheral blood	33	86.8
Bone marrow	5	13.2
**HLA match***		
Match	29	76.3
Mismatch	2	5.3
Haplo	6	15.8
**Conditioning**		
MAC	20	52.6
RIC	18	47.4
**Prophylaxis of acute GVHD**		
Calcineurin-based	37	97.4
mTOR-inhibitor based	1	2.6
**Acute GVHD**		
No	18	47.4
Grade <2	8	21.1
Grade >2	12	31.5
**Delay from tx to sampling**		
< 12 m	14	36.8
12-36 m	22	57.9
> 36 m	2	5.3

AML; acute myeloid leukemia. MDS; myelodysplastic syndrome. NHL; non-hodgkin lymphoma. CLL; chronic lymphocytic leukemia. HL; Hodgkin's lymphoma. MPD; myeloproliferative syndrome. MM; multiple myeloma. MUD; match unrelated donor. Haplo; haploidentical HLA. MAC; myeloablative. RIC; reduced-intensity conditioning. tx; transplantation. *one missing **two missing

Supplementary Table 7. Gene list in the immunogene panel sequencing. Gene names are presented as HUGO Gene Nomenclature Committee (HGNC) symbols.

A2M	A2ML1	ABCB1	ABCF1	ACE	ADA	ADAM10	ADAM17	ADAM8	AGFG1
AICDA	AIMP1	AIRE	ALCAM	ALK	ANP32B	ANPEP	ANXA6	ATM	B2M
BACH2	BANK1	BATF	BAX	BCAM	BCAP31	BCL2	BCL2L1	BCL6	BIRC3
BLK	BLM	BMP2	BSG	BST1	BST2	BTK	BTLA	C1QA	C1QB
C1QBP	C1QC	C1QL1	C1QL2	C1QL3	C1QL4	C1QTNF2	C1QTNF3	C1QTNF4	C1QTNF5
C1QTNF6	C1QTNF7	C1R	C1RL	C1S	C2	C3	C3AR1	C4A	C4B
C4BPA	C4BPB	C5	C5AR1	C6	C7	C8A	C8B	C8G	C9
C9orf47	CAMP	CANX	CASP1	CASP10	CASP2	CASP3	CASP4	CASP6	CASP7
CASP8	CASP9	CCBP2	CCL1	CCL11	CCL13	CCL14	CCL15	CCL16	CCL17
CCL18	CCL19	CCL2	CCL20	CCL21	CCL22	CCL23	CCL24	CCL25	CCL26
CCL27	CCL28	CCL3	CCL3L1	CCL3L3	CCL4	CCL4L1	CCL4L2	CCL5	CCL7
CCL8	CCND2	CCND3	CCNE1	CCR1	CCR10	CCR2	CCR3	CCR4	CCR5
CCR6	CCR6	CCR7	CCR9	CCRL1	CCRL2	CCRN4L	CD101	CD109	CD14
CD151	CD160	CD163	CD164	CD164L2	CD180	CD19	CD1A	CD1B	CD1C
CD1D	CD1E	CD2	CD200R1	CD200R1L	CD207	CD209	CD22	CD226	CD244
CD247	CD248	CD27	CD274	CD276	CD28	CD2AP	CD2BP2	CD300A	CD300C
CD300E	CD300LB	CD300LF	CD300LG	CD302	CD320	CD33	CD34	CD36	CD37
CD38	CD3D	CD3E	CD3EAP	CD3G	CD4	CD40	CD40LG	CD44	CD46
CD47	CD48	CD5	CD52	CD53	CD58	CD59	CD5L	CD6	CD63
CD68	CD69	CD7	CD70	CD72	CD74	CD79A	CD79B	CD80	CD81
CD82	CD83	CD84	CD86	CD8A	CD8B	CD9	CD93	CD96	CD97
CD99	CD99L2	CDH5	CDK6	CDKN1A	CEACAM1	CEACAM3	CEACAM5	CEACAM6	CEACAM8
CEBPB	CEBPE	CFD	CFH	CFHR1	CFHR2	CFHR3	CFHR4	CFHR5	CFI
CFLAR	CFP	CHEK1	CHL1	CHUK	CIITA	CISH	CKLF	CLCF1	CLEC10A
CLEC12A	CLEC16A	CLEC4A	CLEC4C	CLEC4D	CLEC4E	CLEC4M	CLEC5A	CLEC6A	CLEC7A
CLECL1	CLIP1	CLIP2	CLU	CMKLR1	CMTM1	CMTM2	CMTM3	CMTM4	CMTM5
CMTM6	CMTM7	CMTM8	COLEC12	CR1	CR1L	CR2	CRADD	CRLF1	CRLF2
CRLF3	CRP	CSF1	CSF1R	CSF2	CSF2RA	CSF2RB	CSF3	CSF3R	CSN2
CTLA4	CTSG	CTSS	CX3CL1	CX3CR1	CXCL1	CXCL10	CXCL11	CXCL12	CXCL13
CXCL14	CXCL16	CXCL2	CXCL3	CXCL5	CXCL6	CXCL9	CXCR1	CXCR2	CXCR3
CXCR4	CXCR5	CXCR6	CXCR7	CYBA	CYBB	CYSLTR1	CYTL1	DARC	DCD
DCLRE1C	DDR1	DEFA1	DEFA3	DEFA4	DEFA5	DEFA6	DEFB1	DEFB103A	DEFB105A
DEFB106A	DEFB119	DEFB123	DEFB4A	DKC1	DOCK2	DPP4	DUSP1	EBF1	EBF2
EBI3	EGF	EIF2AK2	ELK1	EMR3	ENC1	ENG	ENTPD1	EPO	EPX
ERAP1	ERGIC2	ERLIN1	ETS1	ETS2	F3	FADD	FAK	FAS	FASLG
FCAMR	FCAR	FCER1A	FCER1G	FCER2	FCGR1A	FCGR2A	FCGR2B	FCGR2C	FCGR3A
FCGR3B	FCGRT	FCRL5	FCRLA	FGFR1	FGFR2	FGFR3	FGFR4	FGR	FLT3
FOS	FOXK2	FOXN1	FOXO1	FOXO3	FOXP3	FRK	FUT3	FYN	G6PD
GADD45A	GNLY	GP1BA	GP1BB	GP5	GP9	GPR183	GUSB	GYPA	GYPB
GYPC	GYPE	GZMA	GZMB	GZMK	GZMM	HAMP	HCK	HGF	HLA-A
HLA-B	HLA-C	HLA-DMA	HLA-DMB	HLA-DOA	HLA-DOB	HLA-DPA1	HLA-DPB1	HLA-DQA1	HLA-DQB1
HLA-DQB2	HLA-DRA	HLA-DRB5	HLA-E	HLA-F	HMGB1	HMMR	HPS3	HRAS	HRH2
HRH4	HSP90B1	HSPA4	HSPA6	HSPD1	HTN3	ICAM1	ICAM2	ICAM3	ICAM4
ICOS	IFI16	IFI27	IFI35	IFI44L	IFIH1	IFIT1	IFIT1B	IFIT2	IFIT3
IFITM1	IFNA2	IFNAR2	IFNB1	IFNG	IFNGR1	IGF1R	IGF2R	IGJ	IGLL1
IGSF8	IKBKAP	IKBKB	IKBKE	IKBKG	IKZF1	IKZF2	IKZF3	IL-17	IL10
IL10RA	IL10RB	IL11	IL11RA	IL12A	IL12B	IL12B	IL12RB1	IL12RB2	IL13
IL13RA1	IL13RA2	IL15	IL15RA	IL16	IL17A	IL17B	IL17C	IL17D	IL17F
IL17RA	IL17RB	IL17RC	IL17RD	IL17RE	IL18	IL18BP	IL18R1	IL18RAP	IL19
IL1A	IL1B	IL1F10	IL1R1	IL1R2	IL1RAP	IL1RAPL1	IL1RAPL2	IL1RL1	IL1RL2
IL1RN	IL2	IL20	IL20RA	IL21	IL21R	IL22	IL22RA1	IL22RA2	IL23A
IL23R	IL24	IL25	IL26	IL27	IL27RA	IL28A	IL28B	IL28RA	IL29
IL2RA	IL2RB	IL2RG	IL3	IL31	IL31RA	IL32	IL33	IL36A	IL36G

IL36RN	IL37	IL3RA	IL4	IL4I1	IL4R	IL5	IL5RA	IL6	IL6R
IL6ST	IL7	IL7R	IL8	IL9	IL9R	ILF2	ILF3	INDO	INSR
IRAK1	IRAK1BP1	IRAK2	IRAK3	IRAK4	IRF1	IRF2	IRF4	IRF5	IRF8
IRF9	ISG20	ITFG1	ITGA1	ITGA2	ITGA2B	ITGA3	ITGA4	ITGA5	ITGA6
ITGAD	ITGAE	ITGAL	ITGAM	ITGAV	ITGAX	ITGB1	ITGB2	ITGB3	ITGB4
JAK1	JAK2	JAK3	JUN	KDR	KEL	KIF21B	KIR2DL1	KIR2DL3	KIR2DL4
KIR2DS4	KIR3DL1	KIR3DL2	KIR3DL3	KIT	KITLG	KLRB1	KLRC1	KLRC2	KLRD1
KLRK1	KRAS	L1CAM	LAG3	LAIR1	LAIR2	LAMP1	LAMP2	LAMP3	LAMTOR3
LAX1	LCK	LCP2	LEAP2	LEP	LIF	LIFR	LIG1	LIG4	LILRA1
LILRA2	LILRA3	LILRA4	LILRA4	LILRA5	LILRA6	LILRB1	LILRB2	LILRB3	LILRB4
LILRB5	LITAF	LPO	LRP1	LTA	LTB	LTB4R	LTB4R2	LTBR	LTF
LY75	LY86	LY9	LY96	LYG2	LYN	LYZ	MAF	MAL	MAP2K3
MAP2K4	MAP2K6	MAP3K1	MAP3K14	MAP4K4	MAPK1	MAPK10	MAPK11	MAPK12	MAPK13
MAPK14	MAPK3	MAPK6	MAPK7	MAPK8	MAPK8IP3	MAPK9	MAPKAPK2	MARCO	MASP1
MASP2	MBL2	MBP	MBTPS1	MCAM	MCL1	MDM2	MFI2	MICA	MICB
MIF	MME	MMP9	MPL	MPO	MR1	MRC1	MRC2	MRE11A	MS4A1
MS4A3	MS4A5	MSR1	MST1	MST1R	MTOR	MUC1	MX1	MYC	MYD88
MYLK	NCAM1	NCF1	NCF2	NCF4	NCR1	NCR2	NCR3	NDUFS3	NFATC1
NFATC2	NFATC3	NFATC4	NFIL3	NFKB1	NFKB2	NFKBIA	NFKBIB	NFKBIE	NFKBIL1
NFRKB	NKX2-3	NOD2	NOL3	NOP9	NOS2	NPTN	NRAS	NT5E	PADI4
PAFAH1B1	PAFAH1B2	PAFAH1B3	PAFAH2	PARP1	PDCD1	PDCD1LG2	PDGFB	PDGFRA	PDGFRB
PELI1	PELI2	PF4	PGLYRP1	PGLYRP2	PGLYRP3	PGLYRP4	PIAS3	PIK3CG	PIK3R1
PILRA	PIM1	PLA2G7	PLA2R1	PLAA	PLAUR	PLK3	PLXNC1	PNP	POMC
POU2AF1	PPBP	PPIA	PPP3CA	PPP3CB	PPP3CC	PPP3R1	PPP3R2	PRDM1	PRDX6
PRF1	PRG2	PRKCD	PRKCQ	PRNP	PROCR	PROM1	PRSS16	PSG1	PSIP1
PSMB10	PSMB5	PSMB6	PSMB7	PSMB8	PSMB9	PSME1	PSME2	PSME3	PSMF1
PSTPIP1	PTAFR	PTEN	PTGER4	PTGFRN	PTGS2	PTK2	PTK2B	PTPN11	PTPN2
PTPN22	PTPN6	PTPRC	PTPRCAP	PTPRE	PTPRJ	PVR	PVRL1	PVRL2	PXK
RAC1	RAC2	RAG1	RAG2	RBPJ	REL	RELA	RELB	RFX1	RFX5
RFXANK	RFXAP	RGS1	RHAG	RHCE	RHD	RIPK1	RIPK2	RNASE7	ROR1
RORA	RORC	RPA1	S100A8	SAMSN1	SARM1	SCARB1	SCARB2	SCGB3A1	SDC1
SDF2	SDF2L1	SELE	SELL	SELP	SELPLG	SEMA4D	SEMA7A	SERPING1	SH2B3
SH2D1A	SIGIRR	SIGLEC1	SIGLEC15	SIGLEC5	SIGLEC6	SIRPA	SIVA1	SLA	SLAMF1
SLAMF6	SLAMF7	SLC3A2	SLC44A1	SLC4A1	SLC7A5	SMARCAL1	SOCS1	SOCS2	SOCS3
SOCS4	SOCS5	SOCS6	SOCS7	SOD1	SOD3	SPN	SPP1	SRC	ST6GAL1
STAT1	STAT2	STAT3	STAT4	STAT5A	STAT5B	STAT6	TAGAP	TAL1	TANK
TAP1	TAP2	TAP2	TAPBP	TBK1	TBX21	TCF3	TCF7	TCN2	TDP2
TEK	TFRC	TGFB1	THBD	THY1	TICAM1	TICAM2	TIMP1	TIRAP	TLR1
TLR10	TLR2	TLR3	TLR4	TLR5	TLR6	TLR7	TLR8	TLR9	TLR9
TMED7	TNF	TNFAIP3	TNFRSF10A	TNFRSF10B	TNFRSF10C	TNFRSF10D	TNFRSF11A	TNFRSF12A	TNFRSF13B
TNFRSF13B	TNFRSF13C	TNFRSF14	TNFRSF17	TNFRSF18	TNFRSF1A	TNFRSF1B	TNFRSF25	TNFRSF4	TNFRSF8
TNFRSF9	TNFSF10	TNFSF11	TNFSF12	TNFSF13	TNFSF13B	TNFSF14	TNFSF15	TNFSF4	TNFSF8
TNIP1	TOLLIP	TONSL	TRADD	TRAF1	TRAF2	TRAF3	TRAF3IP1	TRAF4	TRAF5
TRAF6	TRAF7	TSPYL2	TYK2	ULBP1	UNG	WAS	WASF1	WASF3	VCAM1
VCAN	VEGFA	WFDC12	WIPF1	VPREB1	XCL1	XCL2	XCR1	XRCC5	YES
YWHAZ	ZAP70	ZEB1	ZFP36	ZFP36L1					

Supplementary Table 8. Corresponding IMGT nomenclature used for the Vβ segments in IOTest Beta Mark TCR Vβ repertoire Kit.

Nomenclature (IOTest Beta Mark TCR Vβ repertoire Kit)	IMGT nomenclature
Vβ 1	TRBV9
Vβ 2	TRBV20-1
Vβ 3	TRBV28
Vβ 4	TRBV29-1
Vβ 5.1	TRBV5-1
Vβ 5.2	TRBV5-6
Vβ 5.3	TRBV5-5
Vβ 7.1	TRBV4-1. TRBV4-2. TRBV4-3
Vβ 7.2	TRBV4-3
Vβ 8	TRBV12-3. TRBV12-4
Vβ 9	TRBV3-1
Vβ 11	TRBV25-1
Vβ 12	TRBV10-3
Vβ 13.1	TRBV6-5. TRBV6-6. TRBV6-9
Vβ 13.2	TRBV6-2
Vβ 13.6	TRBV6-6
Vβ 14	TRBV27
Vβ 16	TRBV14
Vβ 17	TRBV19
Vβ 18	TRBV18
Vβ 20	TRBV30
Vβ 21.3	TRBV11-2
Vβ 22	TRBV2
Vβ 23	TRBV13

Supplementary Table 9. Primer sets of *mTOR. NFkB2* and *TLR2* amplicon sequencing

Primer name	Sequence
mTOR-F	5’-ACACTCTTTCCCTACACGACGCTCTTCCGATCTTCCCTGTAGTCCCGGATGAG-3’
mTOR-R	5’-AGACGTGTGCTCTTCCGATCTGCCTGTGTTCTGAGCTGCTC-3’
NFkB2-F	5’-ACACTCTTTCCCTACACGACGCTCTTCCGATCTATCCCATTCCTGTCCCCATTT-3’
NFkB2-R	5’-AGACGTGTGCTCTTCCGATCTAGTGCACCTGAGGCTGGG-3’
TLR2-F	5’-ACACTCTTTCCCTACACGACGCTCTTCCGATCTAAGACTTTGGAAGCTGGTGG-3’
TLR2-R	5’-AGACGTGTGCTCTTCCGATCTCGGACATCCTGAACCTGCT-3’

Abbreviations: F. forward primer; R. reverse primer

Supplementary Table 10. Primer set for *mTOR. NFkB2* and *TLR2* capillary sequencing

Gene	Forward primer	Reverse primer	Product length
MTOR	5'-TCTGCCTGTGTTCTGAGCTG-3'	5'-CGATGCTCGATGTTGAGAAG-3'	210
NFkB2	5'-CAACTCCGGATCTCGCTCTC-3'	5'-GGCATGACTCACTGGGTTGT-3'	259
TLR2	5'-GCCTCCCTCTTACCCATGTT-3'	5'-TACCACAGGCCATGGAAACG-3'	368

Supplementary Table 11. List of *mTOR. NFkB2* and *TLR2* mutagenesis primers

Primer name	Sequence
MTOR_P2229R-F	5’-GAGATACGCTGTCATCCGTTTATCGACCAACTCGG-3’
MTOR_P2229R-R	5’-CCGAGTTGGTCGATAAACGGATGACAGCGTATCTC-3’
NFkB2_P882Q-F	5’-TCCCCCAAATCTGGGCCACCATCTCCCGCCCACCC-3’
NFkB2_P882Q-R	5’-GGGTGGGCGGGAGATGGTGGCCCAGATTTGGGGGA-3’
TLR2_W558L-F	5’-CAAAGTCTTGATTGATTTGCCAGCAAATTACCTGT-3’
TLR2_W558L-R	5’-ACAGGTAATTTGCTGGCAAATCAATCAAGACTTTG-3’

Abbreviations: F. forward primer; R. reverse primer

Supplementary Table 12. Primer list of RT-qPCR

Target gene	Sequence
FOS-F	5’-CCGGGGATAGCCTCTCTTACT-3’
FOS-R	5’-CCAGGTCCGTGCAGAAGTC-3’
ELK1-F	5’-AATCGGAAGAGCTTAATGTGGAG-3’
ELK1-R	5’-CTTGGTGGTTTCTGGCACAA-3’
ACTB-F	5’-GTTGTCGACGACGAGCG-3’
ACTB-R	5’-GCACAGAGCCTCGCCTT-3’

Abbreviations: F. forward primer; R. reverse primer
