# Supplementary material for: Somatic mTOR mutation in clonally expanded T lymphocytes associated with chronic graft versus host disease
Source: Nat Commun. 2020 May 7;11:2246. doi: 10.1038/s41467-020-16115-w (PMC7206083; doi:10.1038/s41467-020-16115-w)
Supplement: Supplementary file 3 — Reporting Summary [file 41467_2020_16115_MOESM3_ESM.pdf]

nature research

Corresponding author(s): Satu Mustjoki

Last updated by author(s): Feb 14, 2020

Reporting Summary

Nature Research wishes to improve the reproducibility of the work that we publish. This form provides structure for consistency and transparency in reporting. For further information on Nature Research policies, see Authors & Referees and the Editorial Policy Checklist.

Statistics

For all statistical analyses, confirm that the following items are present in the figure legend, table legend, main text, or Methods section.

n/a	Confirmed	
☐	☒	The exact sample size (*n*) for each experimental group/condition, given as a discrete number and unit of measurement
☐	☒	A statement on whether measurements were taken from distinct samples or whether the same sample was measured repeatedly
☐	☒	The statistical test(s) used AND whether they are one- or two-sided<br>*Only common tests should be described solely by name; describe more complex techniques in the Methods section.*
☐	☒	A description of all covariates tested
☐	☒	A description of any assumptions or corrections, such as tests of normality and adjustment for multiple comparisons
☐	☒	A full description of the statistical parameters including central tendency (e.g. means) or other basic estimates (e.g. regression coefficient) AND variation (e.g. standard deviation) or associated estimates of uncertainty (e.g. confidence intervals)
☐	☒	For null hypothesis testing, the test statistic (e.g. *F*, *t*, *r*) with confidence intervals, effect sizes, degrees of freedom and *P* value noted<br>*Give P values as exact values whenever suitable.*
☒	☐	For Bayesian analysis, information on the choice of priors and Markov chain Monte Carlo settings
☒	☐	For hierarchical and complex designs, identification of the appropriate level for tests and full reporting of outcomes
☒	☐	Estimates of effect sizes (e.g. Cohen's *d*, Pearson's *r*), indicating how they were calculated

Our web collection on statistics for biologists contains articles on many of the points above.

Software and code

Policy information about availability of computer code

Data collection	No software was used for data collection.
Data analysis	GraphPad Prism 8 for Mac OS X (version 8.3.0), R for Mac OS X (version 3.5.1), FlowJo software (Version 10.6.1) and xCELLigence RTCA Software (version 2.1.0, ACEA Biosciences) were used for data analysis. All custom scripts made for scRNA-seq, TCRαβ-seq and healthy data are public to everyone: analyses can be found on https://github.com/janihuuh/gvhd_som_mut.

For manuscripts utilizing custom algorithms or software that are central to the research but not yet described in published literature, software must be made available to editors/reviewers. We strongly encourage code deposition in a community repository (e.g. GitHub). See the Nature Research guidelines for submitting code & software for further information.

Data

Policy information about availability of data

All manuscripts must include a data availability statement. This statement should provide the following information, where applicable:

- Accession codes, unique identifiers, or web links for publicly available datasets
- A list of figures that have associated raw data
- A description of any restrictions on data availability

Data supporting the findings of this work are available within the paper and its Supplementary Information files. The source data underlying Figs. 1b, 2c, 3a, d, e, 5a, c, d, 6a-g, and Supplementary Figs. 3, 4 , 5b, 6b, 9a-d, 10a, b, and 11 are provided as a Source Data file. The TCRB sequence data that support the findings of this study are available in ImmuneAccess with the identifier [https://doi.org/10.21417/DK2020NC]. The scRNA seq from healthy control is available from [https://support.10xgenomics.com/single-cell-gene-expression/datasets/3.1.0/5k_pbmc_protein_v3]. The read counts of scRNA seq data from index patient are provided as source file. Whole-exome sequencing data from the index patient is available from the corresponding author upon suitable request owing to regulations pertaining to the authors ethics permit and deposition of these data in public repositories.

Field-specific reporting

Please select the one below that is the best fit for your research. If you are not sure, read the appropriate sections before making your selection.

☒ Life sciences ☐ Behavioural & social sciences ☐ Ecological, evolutionary & environmental sciences

For a reference copy of the document with all sections, see nature.com/documents/nr-reporting-summary-flat.pdf

Life sciences study design

All studies must disclose on these points even when the disclosure is negative.

Sample size	Sample size calculations were not made. This study started from index patient case and main findings are related to this case. For the screening cohorts we included all patient samples that we were able to have from 3 different institutes.
Data exclusions	No data was excluded.
Replication	For experimental analyses at least 3 independent experiments were performed and results replicated successfully. Sequencing data was confirmed with multiple techniques (exome, targeted panel, amplicon sequencing).
Randomization	Participants were allocated to groups based on the clinical status which was derived from treating clinicians.
Blinding	Investigators were not blinded for group allocation and this was not relevant related to the main objectives of the study.

Reporting for specific materials, systems and methods

We require information from authors about some types of materials, experimental systems and methods used in many studies. Here, indicate whether each material, system or method listed is relevant to your study. If you are not sure if a list item applies to your research, read the appropriate section before selecting a response.

Materials & experimental systems

n/a	Involved in the study	
☐	☒	Antibodies
☐	☒	Eukaryotic cell lines
☒	☐	Palaeontology
☒	☐	Animals and other organisms
☐	☒	Human research participants
☐	☒	Clinical data

Methods

n/a	Involved in the study	
☒	☐	ChIP-seq
☐	☒	Flow cytometry
☒	☐	MRI-based neuroimaging

Antibodies

Antibodies used

Western blotting:
S6 (Cell signaling, Clone: 54D2, Cat#: 2317S, Lot#: 4)
Phospho-S6 (Ser235/236) (Cell signaling, Clone: D57.2.2E, Cat#: 4858T, Lot#: 16)
Akt (Cell signaling, Clone: C67E7, Cat#: 4691T, Lot#: 20)
Phospho-Akt (Ser473) (Cell signaling, Cat#: 9271T, Lot#: 14)
p70S6 kinase (S6K1) (Cell signaling, Clone: 49D7, Cat#: 2708T, Lot#: 7)
p-p70S6 kinase (p-S6K1, Thr421/Ser424) (Cell signaling, Cat#: 9204S, Lot#: 11)
NFkB2 (p100/p52) (Cell signaling, Cat#: 4882S, Lot: 4)
mTOR (Cell signaling, Clone: 7C10, Cat#: 2983, Lot#: 16)
p-4E-BP1 (Cell signaling, Clone: 236B4, Cat#: 2855, Lot#: 26)
4E-BP1 (Cell signaling, Cat#: 9452, Lot#: 12)
Rictor (Cell signaling, Clone: 53A2, Cat#: 2114, Lot#: 7)
Raptor (Cell signaling, Clone: 24C12, Cat#: 2280, Lot#: 13)
phospho-FoxO1 (Thr24)/FoxO3a (Thr32) (Cell signaling, Cat#: 9464, Lot#: 7)
FoxO1 (Cell signaling, Clone: C29H4, Cat#: 2880, Lot#: 11)
FoxO3a (Cell signaling, Clone: 75D8, Cat#: 2497, Lot#: 8)
TSC1 (Cell signaling, Clone: D43E2, Cat#: 6935, Lot#: 3)
TSC2 (Cell signaling, Clone: D93F12, Cat#: 4308, Lot#: 6)
Rabbit IgG Isotype control (Cell signaling, Clone: DA1E, Cat#: 3900, Lot#: 34)
Deptor (Novus Biologicals, Cat#: NBP1-49674SS, Lot#: D2)
beta actin (Abcam, Clone: AC15, Cat#: ab6276, Lot#: GR66278-11)

Multiplexed Immunohistochemistry:
CD3 (Abcam, Clone: EP449E, Cat#: ab52959, Lot#: GR140731)

CD4 (Abcam, Clone: EPR6885, Cat#: ab133616, Lot#: GR218457)
CD8 (BioSB, Clone: C8/144B, Cat#: BSB 5174, Lot#: 5174JDL05)

Flow cytometry:
CD3 (BD Biosciences, Fluorophore: APC, Clone: SK7, Cat#: 345767, Lot#: 7236657)
CD3 (BD Biosciences, Fluorophore: Pe-Cy7, Clone: SK7, Cat#: 557851, Lot#: 8037645)
CD3 (BD Biosciences, Fluorophore: APC, Clone: UCHT1, Cat#: 561810, Lot#: 8316946)
CD4 (BD Biosciences, Fluorophore: PerCP, Clone: SK3, Cat#: 345770, Lot#: 6281605)
CD8 (BD Biosciences, Fluorophore: PE-Cy7, Clone: SK1, Cat#: 335822, Lot#: 8272690)
CD8 (BD Biosciences, Fluorophore: PerCP, Clone: SK1, Cat#: 345774, Lot#: 82152)
CD8 (BD Biosciences, Fluorophore: FITC, Clone: SK1, Cat#: 345772, Lot#: 7235793)
CD16 (BD Biosciences, Fluorophore: PE, Clone: LEU11C, Cat#: 332779, Lot#: 4100654)
CD19 (BD Biosciences, Fluorophore: APC, Clone: SJ25C1, Cat#: 557791, Lot#: 3163945)
CD28 (BD Biosciences, Clone: L293, Cat#: 340975, Lot#: 8277956)
CD45 (BD Biosciences, Fluorophore: PerCP, Clone: 2D1, Cat#: 345809, Lot#: 7079645)
CD45 (BD Biosciences, Fluorophore: APCH7, Clone: 2D1, Cat#: 641417, Lot#: 6265575)
CD45RA (BD Biosciences, Fluorophore: Alexa700, Clone: HI100, Cat#: 560673, Lot#: 7180940)
CD49 (BD Biosciences, Clone: L25, Cat#: 340976, Lot#: 5342638)
CD56 (BD Biosciences, Fluorophore: PE, Clone: NCAM16.2, Cat#: 345812, Lot#: 4113857)
CCR7 (R&D System, Fluorophore: PE, Clone: 150503, Cat#: FAB197P, Lot#: LEU1618031)
Granzyme B (BD Biosciences, Fluorophore: BV510, Clone: GB11, Cat#: 563388, Lot#: 9093962)
TNF-α (BD Biosciences, Fluorophore: V450, Clone: MAb11, Cat#: 561311, Lot#: 8127814)
IFN-γ (BD Biosciences, Fluorophore: V450, Clone: B27, Cat#: 560371, Lot#: 5275825)
β Mark TCR Vβ Repertoire Kit (Beckman Coulter, Cat#: IM3497, Lot#: 66)

Validation

All antibodies are commercially available. The antibodies were validated by the manufacturers (shown on their websites).

Eukaryotic cell lines

Policy information about cell lines

Cell line source(s)

HEK293 from ATCC (Cat#: CRL-1573)
HEK293FT from Thermo Fisher Scientific (Cat#: R70007)

Authentication

Authentication was performed via Promega GenePrint10 System. The result was compared ATCC STR, JCRB STR, ICLC STR database, and DSMZ online STR database. The identity estimates are calculated according to the allele information found in these databases.

Mycoplasma contamination

Cell lines were tested and negative for mycoplasma via MycoAlert Mycoplasma Detection Kit (LONZA, Cat#: LT07-318).

Commonly misidentified lines
(See ICLAC register)

No commonly misidentified cell lines were used.

Human research participants

Policy information about studies involving human research participants

Population characteristics

The blood samples were collected 3 to 102 months (mean 13.5 months) and 2 to 47 months (mean 14.5 months) after allo-HSCT for GvHD and non-cGvHD patients, respectively.

A. Patients with cGvHD

Patient characteristics N (%)

Total 135
- Age at sampling (mean, range) 48 (16-70)
- Patient sex
Female 58 (43,0)
Male 77 (57,0)
- Donor sex*
Female 53 (39,3)
Male 81 (60,0)
- Sex mismatch*
No 73 (54,1)
Yes 61 (45,2)
- Diagnosis
AML, MDS 63 (46,7)
ALL 17 (12,6)
NHL 17 (12,6)
CLL 2 (1,5)
HL 4 (3,0)
MPD 15 (11,1)
MM 11 (8,1)

October 2018

Other   6 (4,4)
- Donor type
Sibling   79 (58,5)
MUD   56 (41,5)
- Stem Cell Source
Peripheral blood 122 (90,4)
Bone marrow 13 (9,6)
HLA match**
Match   114 (84,4)
Mismatch   13 (9,6)
Haplo   7 (5,2)
Conditioning*
MAC   64 (47,4)
RIC   70 (51,9)
- Prophylaxis of acute GVHD
Calcineurin-based 125 (92,6)
mTOR-inhibitor based 10 (7,4)
Acute GVHD**
No   66 (48,9)
Grade <2   17 (12,6)
Grade >2   50 (37,0)
Chronic GVHD*
Mild   28 (20,7)
Moderate-Severe 106 (78,5)
- Delay from tx to sampling
< 12 m   67 (49,6)
12-36 m   53 (39,3)
> 36 m   15 (11,1)

B. Patients without cGVHD

Patient characteristics N (%)

Total   38
- Age at sampling (mean, range) 48 (16-70)
- Patient sex
Female   23 (60,5)
Male   15 (39,5)
Donor sex*
Female   12 (31,6)
Male   26 (68,4)
Sex mismatch*
No   20 (52,6)
Yes   18 (47,4)
- Diagnosis
AML, MDS   23 (60,5)
ALL   6 (15,8)
NHL   2 (5,3)
CLL   2 (5,3)
HL   3 (7,9)
MPD   1 (2,6)
MM   0 (0)
Other   1 (2,6)
- Donor type
Sibling   23 (60,5)
MUD   15 (39,5)
- Stem Cell Source
Peripheral blood 33 (86,8)
Bone marrow 5 (13,2)
HLA match*
Match   29 (76,3)
Mismatch   2 (5,3)
Haplo   6 (15,8)
- Conditioning
MAC   20 (52,6)
RIC   18 (47,4)
- Prophylaxis of acute GVHD
Calcineurin-based 37 (97,4)
mTOR-inhibitor based 1 (2,6)
- Acute GVHD
No   18 (47,4)
Grade <2   8 (21,1)
Grade >2   12 (31,5)
- Delay from tx to sampling
< 12 m   14 (36,8)

	12-36 m 22 (57,9)<br>> 36 m 2 (5,3)<br><br>AML; acute myeloid leukemia, MDS; myelodysplastic syndrome, NHL; non-hodgkin lymphoma, CLL; chronic lymphocytic leukemia, HL; Hodgkin's lymphoma, MPD; myeloproliferative syndrome, MM; multiple myeloma. MUD; match unrelated donor. Haplo; haploidentical HLA. MAC; myeloablative, RIC; reduced-intensity conditioning, tx; transplantation. *one missing **two missing
Recruitment	Samples were collected between 2007-2016 from 135 patients who had developed cGvHD after allo-HSCT (Helsinki University Hospital, Helsinki, Finland, n=8; Turku University Hospital, Turku, Finland, n=37; Hospital de la Princesa, Madrid, Spain, n=19; Hospital Morales Meseguer, Murcia, Spain, n=71). In addition, 38 patients who had not developed cGvHD until the date of sampling, served as a control cohort (Turku University Hospital n=6 and Hospital Morales Meseguer n=32). Additionally, buffy coat samples from 54 healthy blood donors were obtained from the Finnish Red Cross Blood Service.
Ethics oversight	The study was performed in compliance with the principles of Helsinki declaration, and was approved by the ethics committees in the Helsinki University Hospital (Helsinki, Finland), Turku University Hospital (Turku, Finland), Hospital de la Princesa (Madrid, Spain) and Hospital Morales Meseguer (Murcia, Spain).

Note that full information on the approval of the study protocol must also be provided in the manuscript.

Clinical data

Policy information about clinical studies

All manuscripts should comply with the ICMJE guidelines for publication of clinical research and a completed CONSORT checklist must be included with all submissions.

Clinical trial registration	NA
Study protocol	NA
Data collection	NA
Outcomes	NA

Flow Cytometry

Plots

Confirm that:

- [x] The axis labels state the marker and fluorochrome used (e.g. CD4-FITC).
- [x] The axis scales are clearly visible. Include numbers along axes only for bottom left plot of group (a 'group' is an analysis of identical markers).
- [x] All plots are contour plots with outliers or pseudocolor plots.
- [x] A numerical value for number of cells or percentage (with statistics) is provided.

Methodology

Sample preparation	Peripheral blood was collected in sodium heparin Vacutainer tubes (BD Biosciences). Peripheral blood mononuclear cells (PBMCs) were separated from whole blood using density gradient centrifugation with Ficoll-PaqueTM PLUS (GE Healthcare).
Instrument	FACSVerse (BD Biosciences) and BD Accuri™ C6 Plus (BD Biosciences) were applied to analyze for phenotyping T cell subsets and TCR Vβ families. Stained cells were physically isolated by FACs AriaIII (BD Biosciences).
Software	Collection: BD FACSuite Software (version 1.0.6)<br>Analysis: Flowjo (Version 10.6.1)
Cell population abundance	One million cells were stained in 300 uL for flow cytometry to analyze. Purity of sorted cells isolated by FACs AriaIII (BD Biosciences) was more than 99% and verified with the same system.
Gating strategy	Applied gating strategy is described in the supplemental information.

- [x] Tick this box to confirm that a figure exemplifying the gating strategy is provided in the Supplementary Information.
